# Data-Driven Prediction and Design of bZIP Coiled-Coil Interactions

**DOI:** 10.1371/journal.pcbi.1004046

**Published:** 2015-02-19

**Authors:** Vladimir Potapov, Jenifer B. Kaplan, Amy E. Keating

**Affiliations:** 1 Department of Biology, Massachusetts Institute of Technology, Cambridge, Massachusetts, United States of America; 2 Department of Biological Engineering, Massachusetts Institute of Technology, Cambridge, Massachusetts, United States of America; University of Heidelberg, GERMANY

## Abstract

Selective dimerization of the basic-region leucine-zipper (bZIP) transcription factors presents a vivid example of how a high degree of interaction specificity can be achieved within a family of structurally similar proteins. The coiled-coil motif that mediates homo- or hetero-dimerization of the bZIP proteins has been intensively studied, and a variety of methods have been proposed to predict these interactions from sequence data. In this work, we used a large quantitative set of 4,549 bZIP coiled-coil interactions to develop a predictive model that exploits knowledge of structurally conserved residue-residue interactions in the coiled-coil motif. Our model, which expresses interaction energies as a sum of interpretable residue-pair and triplet terms, achieves a correlation with experimental binding free energies of R = 0.68 and significantly out-performs other scoring functions. To use our model in protein design applications, we devised a strategy in which synthetic peptides are built by assembling 7-residue native-protein heptad modules into new combinations. An integer linear program was used to find the optimal combination of heptads to bind selectively to a target human bZIP coiled coil, but not to target paralogs. Using this approach, we designed peptides to interact with the bZIP domains from human JUN, XBP1, ATF4 and ATF5. Testing more than 132 candidate protein complexes using a fluorescence resonance energy transfer assay confirmed the formation of tight and selective heterodimers between the designed peptides and their targets. This approach can be used to make inhibitors of native proteins, or to develop novel peptides for applications in synthetic biology or nanotechnology.

## Author Summary

Protein interactions are important for all life processes, and an ability to rationally control or selectively inhibit protein complexes would impact studies of cellular structure, biological information processing, molecular regulatory processes and other phenomena. Rational protein design has applications in developing biotherapeutics and in advancing synthetic biology and nanotechnology. Rational protein-interaction design has been approached in many ways over the past decades, but it remains a challenge. Computational methods require models to predict binding and tools for applying predictive models in design. Many such methods are based on modeling and evaluating protein structure using physical or semi-physical energy terms. In this work, we used a different strategy, deriving a binding model that describes the protein-protein interactions of basic-region leucine-zipper (bZIP) transcriptions factors directly from a large body of experimental interaction data. Our model showed much better performance than previously published predictors. We used our model, in conjunction with a protein-design strategy that builds new proteins from modular parts of known proteins, to successfully design novel bZIP-like protein domains. We demonstrated experimentally that the designed proteins bind tightly and specifically to a number of human bZIPs that regulate important processes including stress responses and oncogenesis.

## Introduction

The basic-region leucine-zipper (bZIP) transcription factors pose a compelling problem to scientists interested in protein-protein interaction specificity. Dimerization of bZIPs, which is required for high-affinity DNA binding, is mediated by a coiled-coil motif in which fewer than 50 residues per protein chain adopt a structure consisting of two supercoiled alpha helices. The coiled coil can be a homodimer or a heterodimer. Strikingly, this simple motif is sufficient to encode intricately complex interaction patterns among pairs of bZIP family members. For example, biochemical assays using highly purified components have established that 53 human bZIP coiled coils span at least 38 distinct interaction profiles, defined by the strength of a protein’s binding to each of 53 possible interaction partners [1]. This observation demonstrates that an enormous amount of interaction information is encoded compactly in coiled-coil sequences. In fact, Grigoryan et al. conservatively estimated that at least ~1,900 distinct interaction profiles can be encoded by short bZIP-like coiled-coil sequences [2]. A fundamental challenge for computational structural biologists is to decipher the code by which sequence determines interaction selectivity, and the bZIP proteins present an outstanding opportunity to tackle this problem. This is not only important for understanding bZIP proteins and how they control transcription. Because many other interaction domains mediate specific interactions that broadly influence biology, developing methods that allow us to interpret, predict and manipulate the sequence-interaction code of proteins is a compelling objective.

Decades of study have revealed key relationships between coiled-coil sequence and structure and have guided the design of synthetic coiled coils [3–10]. The coiled-coil dimerization motif of the bZIP proteins has a characteristic 7-residue repeat denoted [**abcdefg**]_n_ that defines the placement of residues in each helix relative to the interaction interface. The hydrophobic core is formed by residues at the **a** and **d** heptad positions and is crucial for stability, whereas residues at **g** and **e** positions are usually polar or charged and contribute to specificity. The bZIP dimerization domains belong to the leucine-zipper class of coiled coils, in which core **d** positions are very frequently occupied by leucine. More variable **a**-position sites accommodate polar residues that influence coiled-coil interaction specificity, oligomerization state and helix orientation. bZIPs bind DNA as dimers formed by helices that are aligned in a parallel orientation. Excellent reviews summarize what is known about the influence of sequence on coiled-coil structure [11–13].

Given the simplicity of the coiled coil relative to other protein structures, a fundamental goal is to develop rules or algorithms that can predict coiled-coil structure and interactions from sequence. The general problem has proven extraordinarily difficult due to the large number of related coiled-coil structures that differ in helix number, alignment or orientation. However, impressive progress has been made elucidating determinants of the coiled-coil interaction specificity of the bZIPs. Key insights came from the work of Vinson and colleagues, who quantified the interaction energies of 61 residue-residue pairs that influence bZIP homo- vs. heterodimerization using double-mutant cycles [5,6]. These interaction energies alone can explain many of the dimerization preferences of bZIP proteins, and the availability of experimental coupling energies has supported the development of rule-based schemes for predicting bZIP interactions. Experimental coupling energies have also been used as input, along with other data, to train a machine-learning model for predicting coiled-coil interactions, as discussed further below. Models based on predicting and evaluating the energetics of structures of coiled coils have been markedly less successful than methods that take advantage of experimental interaction data [14–16].

Models or rules for predicting coiled-coil interactions have been evaluated using sparse literature interaction data, including data that describe the effects of mutations in model systems [6,17]. In 2003, a report testing 1,225 pair-wise interactions among human bZIP coiled coils using protein microarrays provided a data set that allowed more systematic testing, and the performance of several prediction schemes was assessed using these results [1,14,16]. Recently, Reinke et al. dramatically expanded both the quantity and quality of experimental bZIP interaction data as part of a study of the evolution of protein interaction specificity [18]. 194 bZIP proteins were identified in seven animal species, and binding affinities were experimentally determined for 5,271 pairs of proteins using a solution fluorescence resonance energy transfer (FRET) assay. These data provided a new opportunity to benchmark the performance of existing prediction methods and develop new modeling approaches. In this paper, we report a machine-learning scheme with prediction accuracy that approaches the estimated reliability of the experimental data, and show how it can be used to design novel and specific peptide binders for four human bZIP targets.

## Results

### Benchmarking

To benchmark prediction methods, we took the experimental bZIP interaction data of Reinke et al. and developed two prediction tests. In one, we compared the experimentally determined K_d_ values for interactions with 1 nM < K_d_ < 5,000 nM to predicted scores. We report the Pearson correlation coefficient R for this test. For the other, we divided the data into strong interactions (K_d_ < 250 nM) and weak/non-interactions (K_d_ ≥ 5,000 nM) and measured the area under the curve for a receiver-operating characteristic (ROC) curve reporting the ability of each method to classify bZIP pairs in the right category. To determine the best performance that we could expect from a computational model in the correlation test, we examined the correlation between experimental K_d_ values measured in duplicate or closely similar experiments in the experimental work (see Methods). This gave R = 0.82–0.86, depending on the test, which is consistent with the error estimated in the original report [18]. We consider this as the maximum achievable prediction accuracy for any computational method on this test.

We tested four published computational methods developed for predicting parallel coiled-coil dimer stability (Table 1). The models that we refer to as Vinson/CE [5,6,16], Fong/SVM [14], and bCIPA [15] take aligned pairs of protein sequences as input and do not require modeling of protein structures. All three models use coupling energies describing **a**_**i**_-**a*'***_**i**_ and **g**_**i**_-**e*'***_**i+1**_ interactions measured for residues in a coiled-coil model system by Vinson and colleagues (here the subscript represents a heptad index and a prime indicates a position on the opposing helix), and some use more extensive amounts of experimental data about coiled-coil interactions. Thus, we refer to these methods as data-derived predictors [5,6]. The HP/S/C model is a hybrid model that uses the Vinson experimental coupling energies in conjunction with structure-based modeling of other residue-residue interactions [16]. We also tested two general-purpose scoring functions that were not developed specifically for coiled coils but can be applied to evaluate modeled coiled-coil structures: dDFIRE [19] and Rosetta [20]. We generated models for each complex using Rosetta, as described in the Methods. There are many ways to score complexes using Rosetta, and although we could not try all possible protocols, we tested a variety of methods and report here the best results obtained.

**Table 1:** Benchmark of existing methods for predicting coiled-coil specificity.

Method	Pearson correlation coefficient, R (948 interactions)	Area under ROC curve (699 weak/non vs. 3,601 strong interactions)
**Data-derived methods**		
Fong/SVM	0.47	0.83
bCIPA	0.38	0.81
Vinson/CE^a^	0.34	0.86
**Hybrid method**		
HP/S/C	0.26	0.79
**Structure-based methods**		
dDFIRE	0.28	0.76
Rosetta	0.13	0.65

Experimentally determined K_d_ values were compared to predicted scores using two tests: the Pearson correlation coefficient (R) and the area under the curve (AUC). The Pearson correlation coefficient is reported only for interactions with 1 nM < K_d_ < 5,000 nM. In the AUC test, the interactions were divided into two classes: strong interactions (K_d_ < 250 nM) and weak/non-interactions (K_d_ ≥ 5,000 nM). The number of interactions used in each test is given in parentheses at the top of each column.

^a^ Vinson and colleagues measured coupling energies for pairs of residues in **a_i_**-**a*'*_i_** and **g_i_**-**e*'*_i+1_** positions. An additional empirical coupling energy of -2 kcal/mol for Leu-Leu interaction at **d_i_**-**d*'*_i_** positions was added to account for these strongly stabilizing interactions, as in [16].

From our benchmarking studies, it was clear that the data-derived models, which incorporate experimental knowledge about coiled-coiled interactions not included in the Reinke et al. data, outperformed the structure-based models (Table 1). The support vector machine developed by Fong et al. was among the most successful predictors both in terms of the linear correlation (R = 0.47) and the area under the curve (AUC = 0.83) for binary classification of tight binding/non-binding. All of the data-derived models, including Fong/SVM, rely heavily on experimental coupling energy data. In fact, the Vinson/CE model, which is a simple sum of experimental coupling energies supplemented with a single empirical term, provides impressive discrimination of high affinity coiled coils from non-interacting pairs of proteins (AUC = 0.86).

### Machine-learning model

**Fig 1:**
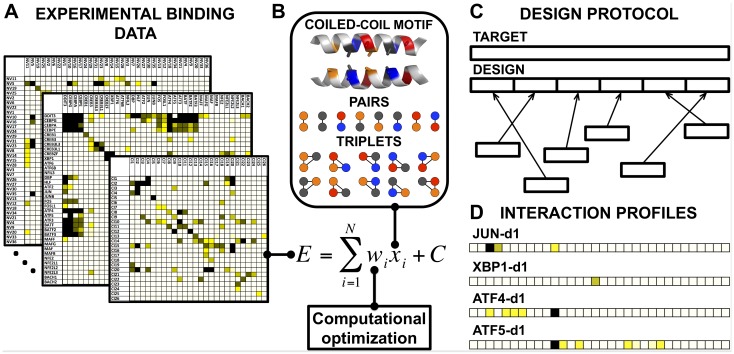
Schematic representation of the model-building and design protocol used to generate selective bZIP-interacting peptides. (A) Experimentally determined dissociation constants for 4,549 bZIP interaction pairs were used as input to train a scoring model. (B) Weights corresponding to contributions from pairs and triplets of residues were fit to experimental binding data using a regression technique. The appropriate optimized weights can be summed to provide a predicted binding energy for two aligned bZIP coiled-coil sequences. (C) Binders were designed by using coiled-coil heptads as building blocks. Optimal combinations of heptads to construct tight-binding and selective designs were identified using integer linear programming in conjunction with the developed scoring function. (D) Designed sequences exhibit tight and selective binding to target bZIP coiled coils. Each square in the cartoon corresponds to a native human bZIP coiled coil, and cells are colored by the strength of interaction of each bZIP with the indicated designed peptide; darker shades correspond to stronger binding. Names of the bZIP proteins corresponding to each cell are given in S5 Table.

The benchmark testing described above indicated that experimental interaction data were essential for generating the models with the highest prediction accuracy. Given the availability of a large amount of new data, we decided to take a machine-learning approach to generate an improved predictor. Following the strategy of Fong et al., we used the conserved coiled-coil structure to pre-define sets of residues that potentially contribute to inter-chain interactions in parallel coiled-coil dimers (see Methods; Fig. 1). Whereas Fong et al. considered only residue pairs, we considered both pairs and triplets of residues. Each pair or triplet was given a weight that was determined using semi-quantitative support vector regression (SemiSVR) to optimize the correlation of predicted scores with experimental data. Our approach was similar to that of Shao et al. and is described in the Methods [21]. The final predicted interaction score for a coiled-coil pair was calculated as a sum over pair and triplet term weights for interactions present in an aligned pair of bZIP sequences.

The experimental data used to train our model came in two forms: binder data, where experimental binding data were fit to determine dissociation constants, and non-binder data, where the experimental data indicate that two proteins interact weakly, if at all (K_d_ ≥ 5,000 nM). Binders and non-binders comprise 20% and 80% of the experimental data, respectively. The flexible optimization framework made it possible to use both types of data when training the model, which was previously reported to improve prediction performance for PDZ domain-peptide interactions [21].

We tested the performance of our model on the combined experimental data, using a rigorous nested cross-validation protocol to assess expected performance (see Methods). We obtained a Pearson correlation coefficient of R = 0.68 and an AUC of 0.94 (Table 2). Noting that the correlation between experimental K_d_ values measured twice for the same set of interactions is in the range R = 0.82–0.86, our model achieves good prediction accuracy, especially considering that we do not treat any atomistic structural details. We also divided all available experimental data into non-overlapping and minimally similar training and test sets: 98% of coiled coils in the training set shared less than 90% sequence identity with coiled coils in the test set when considering residues at **a**, **d**, **e** and **g** positions. Prediction accuracy similar to that in the nested cross-validation assessment was obtained for this test (S1 Table). It is worth noting that in cross-validation, 92% of the coiled coils in the top-level partitions shared less than 90% sequence identity to the coiled coils in any other partitions (see Methods for details on partitioning the dataset). As an additional computational test of the cross-validation procedure and an assessment of possible overfitting, we randomly reshuffled K_d_ values associated with the coiled coils and re-optimized the model on the randomized dataset. As expected, the obtained model had zero correlation and AUC of 0.5 in the nested cross-validation. Finally, we estimated a 95% confidence interval for the obtained Pearson correlation coefficient as 0.68 ± 0.04 by using resampling with replacement 1,000 times on the predicted K_d_ values from the nested cross-validation.

**Table 2:** Performance of predictive models using different sets of residue interactions.

Model	Number of features	Cross-validated performance	
		R	AUC
pairs & triplets	17,239 (829)	0.68 (0.67)	0.94 (0.93)
pairs (all)	1,930 (352)	0.65 (0.64)	0.92 (0.92)
pairs (**a**_**i**_-**a*'***_**i**_, **d**_**i**_-**d*'***_**i**_, **g**_**i**_-**e*'***_**i+1**_)	513 (112)	0.58 (0.58)	0.90 (0.89)

The predictive models were optimized using the combined set of interactions and evaluated with nested cross-validation as described in the Methods. The residue interactions used to describe the coiled-coil interaction were varied as descried in the text. We also applied recursive feature elimination to find a smaller subset of key features that retained good prediction performance. The reduced number of features, and the model performance with this number of features, are given in parentheses.

The rigorous cross-validation procedure that we employed in this study was designed to produce a reliable estimate of generalized model performance assuming that all studied proteins fall into the same overall sequence space. We also assessed performance when the model was trained and tested on two datasets with low sequence identity. We divided the data into a larger set (3182 interactions; 70%) and a smaller set (1367 interactions; 30%) such that no two proteins in the two sets had more than 50% sequence identity. The performance of the model trained on the larger set and tested on the smaller set was decreased relative to the cross-validation tests. The Pearson coefficient for binders was R = 0.50. The easier binary discrimination test produced results similar to those obtained using cross-validation (AUC = 0.90). Decreased performance is expected for the correlation test. When proteins in the training and test sets are very different, the model has limited knowledge about the contributions of many specific residue-residue interactions. This result has important implications for designing new bZIP binders using this type of scoring function: design must be performed in a sequence space similar to the space that was used to train the model.

Considering triplets in addition to pairs of residues improved performance modestly, from R = 0.65 to 0.68. We also considered a model that included only **a**_**i**_-**a*'***_**i**_, **d**_**i**_-**d*'***_**i**_ and **g**_**i**_-**e*'***_**i+1**_ pairs, similar to the models based on experimental coupling energies. The simplified model, with 513 weights, gave R = 0.58. Although adding triplets increased the number of features in the model dramatically, not all sets of pairs and triplets were equally important for accurate prediction performance. The optimized weights for many residue combinations were close to zero. Applying recursive feature elimination allowed us to arrive at a model with 829 pairs and triplets of residues that gave performance as good as the complete model (Methods). Table 2 summarizes the performance of different models derived using SemiSVR.

**Fig 2:**
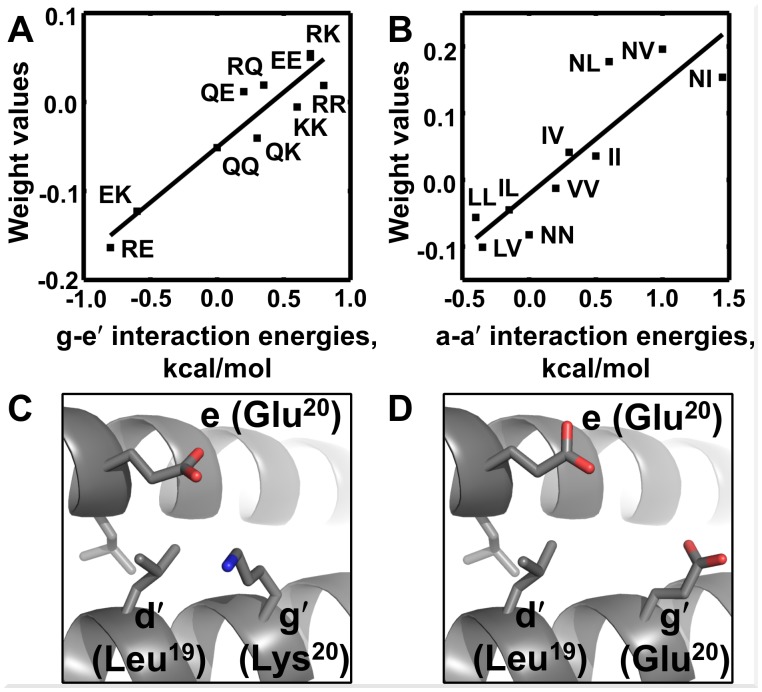
Interpretation of the model weights. Optimized weights correlate with experimentally measured coupling energies reported in the literature: (A) **g**-**e*'*** *R*_*ge*_ = 0.94 (*p* = 5x10^–5^) and (B) **a**-**a*'*** *R*_*aa*_ = 0.89 (*p* = 6x10^–4^) [5,22,23]. (C, D) Examples illustrating triplets of residues that are predicted to be stabilizing (C) or destabilizing (D) according to the derived model. PDB ID 4DMD was used to illustrate the triplets; the structure in panel (D) was obtained by modeling glutamic acid at position 20 using the SCWRL4 side-chain prediction program [57].

The weights in our linear additive model can be interpreted as the contributions of specific pairs or triplets of residues to the predicted stability of a coiled-coil interaction. Interestingly, the optimized weights for many residue pairs agree extremely well with previously measured **a**_**i**_-**a*'***_**i**_ and **g**_**i**_-**e*'***_**i+1**_ coupling energies. Krylov et al. reported **g**_**i**_-**e*'***_**i+1**_ coupling energies for glutamate, glutamine, arginine and lysine residues, and the corresponding **g**_**i**_-**e*'***_**i+1**_ model weights strongly correlate with the experimental values (R = 0.94, n = 10, p = 5 x 10^–5^; Fig. 2) [5,22]. Coupling energies for **a**_**i**_-**a*'***_**i**_ interactions have been reported by two groups, and our weights show a very good correlation with values reported by Steinkruger et al. (R = 0.89, n = 10; p = 6 x 10^–4^; Fig. 2) [6,23]. This contrasts with the difficulty of predicting these weights using simple structure-based models [16]. The apparent interpretability of weights learned using the SemiSVR method is a benefit of our simple encoding of the prediction problem as a linear sum of protein features.

Analysis of the triplets that contributed most to model performance showed some trends. In some triplets, additional stabilization (destabilization) arose from favorable (unfavorable) interactions between residues in adjacent **a**_**i**_-**a*'***_**i**_ and **d**_**i**_-**d*'***_**i**_ layers of the coiled-coil core, e.g. Leu-Leu-Ile and Leu-Val-Ile at **d**_**i**_-**d*'***_**i**_-**a**_**i+1**_ and Ile-Ala-Leu at **a**_**i**_-**a*'***_**i**_-**d**_**i**_ had favorable triplet weights. In other examples, a hydrophobic residue at a **d** heptad position formed favorable contacts with **g**_**i**_-**e*'***_**i+1**_ residue pairs that can form salt bridges (a stabilizing interaction; Fig. 2C). Conversely, with two residues of the same charge at the **g**_**i**_ and **e*'***_**i+1**_ positions, a hydrophobic residue at the **d** position gave a strongly destabilizing triplet, possibly because in structures with this combination of residues the **d** site is exposed to solvent (Fig. 2D).

Our model is based on a large experimental data set and shows significantly improved performance over previously developed models. To assess whether yet more experimental data could further improve prediction performance, we repeated the SemiSVR optimization using between 10% and 100% of all available experimental data and tested performance using cross-validation. Model accuracy improved sharply up to a point, after which it plateaued (S1 Fig.). This suggests that more experimental data of the same type are not likely to improve prediction performance further.

Shao et al. reported that incorporating information about non-interacting pairs improved the prediction accuracy of a SemiSVR method trained to predict PDZ domain-peptide interactions [21]. In our data set, bZIP non-interacting pairs (pairs with K_d_ ≥ 5,000 nM) constituted 80% of the experimental data used for training. We examined whether including these pairs improved our results by training the model using either the binders only (948 interactions) or binders and non-binders together (4,549 interactions total). In both cases, comparing experimental K_d_ values to predicted scores gave the same result: R = 0.67 using only binders and R = 0.68 using binders and non-binders. Thus, incorporating non-interactions in the training set did not improve the correlation with experimentally measured affinities for strongly interacting bZIPs (1 nM ≤ K_d_ < 5,000 nM).

To understand why the non-binder data did not improve predictions for binders (Pearson correlation coefficient), we examined **a**_**i**_-**a*'***_**i**_, **d**_**i**_-**d*'***_**i**_ and **g**_**i**_-**e*'***_**i+1**_ pairwise interactions in binders vs. non-binders (S2 Fig.). The most notable difference was observed for **a**_**i**_-**a*'***_**i**_ interactions. At these positions, Asn-Leu, Asn-Ile and Asn-Val pairs were very frequent for non-binders but rare in binders. Also, **g**_**i**_-**e*'***_**i+1**_ pairs in non-binders tended to have a larger number of repulsive charged interactions (Arg-Arg, Arg-Lys, Glu-Glu), compared to binders. The above-mentioned Asn-Leu, Asn-Ile and Asn-Val pairs are known to strongly destabilize bZIP coiled coils, and this is readily learned when the scoring function is trained (these pairs are assigned strongly unfavorable weights). It is likely that the existence of these strong negative design elements, which simplifies binary discrimination between strong and weak binders, limits the usefulness of the non-binders for improving affinity ranking of binders.

Using non-binder data significantly improved discrimination of strong interactions vs. weak/non-interactions: the AUC dropped from 0.94 to 0.84 when non-interactions were excluded from the training set. This result is important, given that the ability to distinguish between strong versus weak/non-binders is crucial for designing specific protein interactions, as we describe in the next section.

### Designing specific binders

Attractive features of our model, coupled with good performance in prediction benchmarks, prompted us to test it in computational protein design. Our goal was to engineer new coiled-coil peptides that could bind selectively to bZIP domains from human proteins. Because our linear additive model does not require structural modeling of complexes, but instead operates on the sequences of two bZIP proteins, scoring is extremely fast. This is a benefit for exploring large sequence spaces and for considering multiple competing interaction states, as is necessary when designing selective, synthetic bZIP-like peptides.

The design of bZIP-targeting peptides was previously accomplished using the HP/S/C scoring model combined with a novel optimization approach to handle the tradeoff between interaction affinity and interaction specificity with respect to non-target bZIPs that we refer to as “off-targets” [2,24]. Successful designs were obtained for a range of human bZIP targets, as judged by qualitative high-throughput coiled-coil microarray experiments plus circular dichroism spectroscopy assays. In this work, we explored a new strategy for designing bZIP-binding peptides that took advantage of our improved scoring function. We designed novel proteins by assembling them from short heptad sequence fragments derived from natural bZIP proteins (Fig. 1). We reasoned that constraining our designs to a sequence space that closely resembled that of the native proteins for which our prediction algorithm showed good performance would improve our chances of success. Furthermore, residue combinations found in native bZIPs have survived evolutionary selection for function and thus provide conservative design choices at positions where our model is uninformative. This is particularly relevant for solvent-facing **b**, **c** and **f** position residues that do not contribute to the scores predicted by our model. Working within a space of sequences built from native heptads, we optimized the predicted interaction of a design with a bZIP target coiled coil while simultaneously accounting for and disfavoring up to 51 other possible but undesired competing complexes.

The sequence space that we chose for design was very large. Our library of 7-residue fragments included 1,303 heptads from 440 proteins. For a bZIP coiled-coil motif of average length (42 residues = 6 heptads), the total search space was ~5 x 10^18^ possible sequences. It was not feasible to evaluate each of these possible sequences for interaction with each of 51 native human bZIP proteins, even using our fast scorer. Instead, our design procedure used integer linear programming (ILP). ILP is an optimization technique used to find the minimum of an objective function subject to a set of constraints. In our case, we minimized the interaction score between the target and a designed sequence. The design variables were sequence heptads. During minimization, we imposed two types of constraints: (1) the score for the designed sequence binding to off-targets was required to be above a certain value, and (2) the amino-acid composition of the designed sequence was restricted, e.g. we limited the number of polar residues allowed in the core positions. Full details of the optimization protocol are provided in the Methods.

We chose five human bZIP proteins as targets: X-box binding protein 1 (XBP1), JUN, Activating Transcription Factor (ATF) 3, ATF4 and ATF5. These are proteins for which an interaction partner was designed in prior work, but either the affinity or the specificity of the design left room for improvement [2,25]. XBP1 forms a strong homodimer (K_d_ = 8 nM), is important for regulating the unfolded protein response and plasma cell differentiation, and has been implicated as an important gene contributing to breast cancer progression [26–28]. XBP1 can also form coiled-coil heterodimers with CREBZF, ATF6 and ATF6B [18]. JUN, ATF3, ATF4 and ATF5 do not homodimerize strongly via their coiled-coil regions but can form many strong heterodimers with other bZIP transcription factors [1,18,29]. These four proteins play important roles in stress responses, tissue differentiation, and the unfolded protein response. Developing reagents that can selectively target bZIPs, and thus inhibit their DNA binding function, may be useful for further elucidating biological function and for testing the potential of bZIPs as therapeutic targets [30–35]. We designed nine 42-residue long sequences targeting the coiled-coil regions of XBP1, JUN, ATF3, ATF4 and ATF5, using the protocol described in the Methods (Table 3). Designed peptides were tested for binding to human bZIP constructs that included the DNA-binding and coiled-coil domains of each target [18].

**Table 3:** Binding of designed peptides to their bZIP targets.

Design	Target	K_d_ at 37°C (nM)	Designed sequence
XBP1-d1 ^1^	XBP1	172 ± 65	ereaqlenrvahlkeknqelkaqnlhlkealseaqnrnqelknda
XBP1-d2	XBP1	≥ 5,000	aetdqlenrvkdlkkkneslkeekrqasnkykalltnnrslkvka
XBP1-d2* ^2^	XBP1	≥ 5,000 (269 at 23°C)	aetdqlenrvkdlkkkneslkeekrqa**k**nk**l**kalltnnrslkvka
JUN-d1 ^1^	JUN	5.8 ± 0.7	siaatlekeeanlekmnkklaaeiesllkekdklesvlnyhe
JUN-d2	JUN	1	qralqlqkekerlekmnkklaaeieslleererlesvlnyhe
ATF3-d1	ATF3	~1,000	ndlarlenkaeelkvqnrilvderkylqreiselhdelaahe
ATF3-d2	ATF3	564	nlvaqlekknealkaenaaleieriqlqdkieelkyelaaie
ATF3-d3	ATF3	≥ 5,000 (117 at 4°C)	kdaaslenkkeelkvqnrilvderkylqmmnselkdelaahe
ATF4-d1 ^1^	ATF4	9.3 ± 2.3	nqiktlrtrlsklrkdnlqlekdianlerkakdlraekeqleyel
ATF5-d1 ^1^	ATF5	1,680 ± 1,050	kriaylrqriaelrnenhvlesriqrmekekdalqqdrdhleyel
ATF5-d1 ^3^	ATF4	4.9 ± 1.0	(same as above)

^1^ These four designs were chosen for further characterization.

^2^ A double mutant of XBP1-d2, in which serine at position **4e** and tyrosine at position **5a** were mutated to lysine and leucine, respectively.

^3^ Although ATF5 was used in the computational design, binding was tighter to ATF4, which shares 69.2% sequence identity with ATF5 in ***a***, ***d***, ***e*** and ***g*** positions.

To determine whether the designed peptides could interact with their intended partners, designs were labeled with rhodamine at the C-terminus and tested in a direct-binding FRET assay against their C-terminally fluoresceinated bZIP target [18]. Eight of nine designs bound to their target with K_d_ < 5,000 nM at one or more of the temperatures tested (4, 23, or 37°C). One additional design, JUN-d3, was not expressed in sufficient yields for testing. After several attempts to improve expression, this design was not pursued further. Design XBP1-d2 did not bind detectably to XBP1. However, a double mutant of XBP1-d2, in which a serine at **4e** and a tyrosine at **5a** were mutated to lysine and leucine, respectively, bound XBP1 with a K_d_ of 269 nM at 23°C. These mutations were chosen based on the residues in equivalent positions in the successful design XBP1-d1. Lysine at **4e** can potentially interact favorably with glutamic acid at **4g** of XBP1, and leucine at **5a** is a frequently observed residue in **a** positions that might form stabilizing hydrophobic contacts with threonine at **5a** in XBP1. The predicted interaction score for the double mutant was very similar to the score for the original XBP1-d2 design.

**Fig 3:**
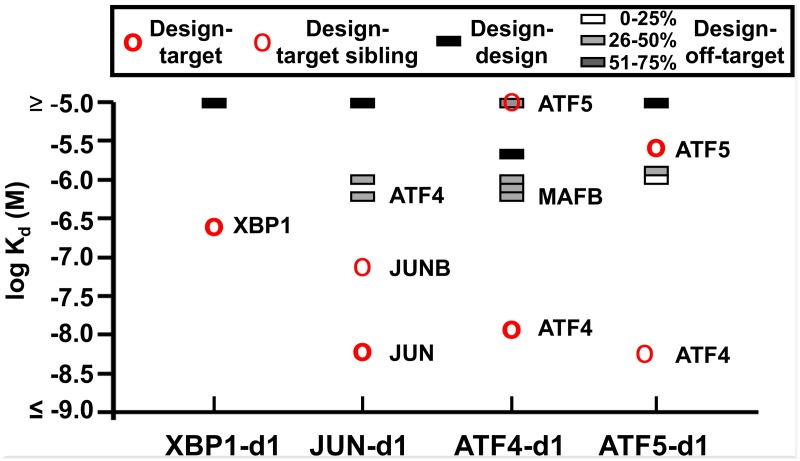
Specificity profiles of four designs tested at 37°C. The designed binders interact strongly with their targets as rhodamine-labeled peptides (bold red circles). Thin red circles show interactions with other bZIPs in the same family as the target. The ATF5-d1 design bound more tightly to ATF4 (thin red circle) than to ATF5. The designed proteins do not form strong homodimers (black bars), and there are large specificity gaps between the design/target interactions and design/off-target interactions (white and grey bars, colored according to target-off-target sequence identity at **a**, **d**, **e** and **g** positions). All K_d_ values are listed in S5–S12 Tables.

The four peptides with the highest target-binding affinities were chosen for further characterization (Table 4). Designs against XBP1, JUN, ATF4 and ATF5 bound their targets with K_d_ values of 172 nM, 6 nM, 9 nM, and 1680 nM, respectively (Fig. 3). Analysis of 1:1 mixtures of these four designed peptides with their targets by analytical ultracentrifugation gave molecular weights consistent with the formation of heterodimers and not higher order species (Methods, S2 Table and S3 Fig.). During the design process, family members of the target were not treated as off-targets, because interactions with closely similar proteins were judged to be difficult to avoid in most cases. Indeed, we observed interactions of the designed peptides with target family members in some cases. For example, ATF5-d1 bound more tightly to ATF4 (K_d_ = 4.9 nM) than to ATF5. Binding of ATF5-d1 to ATF4 was also predicted to be more favorable than interaction with ATF5 by our model (scores of 0.75 vs. 0.40). ATF4 has 69% sequence identity to ATF5 in the core **a**, **d**, **e** and **g** positions.

**Table 4:** Molecular weights of designed bZIP complexes determined by analytical ultracentrifugation.

Design	Target	K_d_ at 37°C (nM)	Lowest off-target K_d_ at 37°C (nM)	Ratio of fitted to expected MW for a 1:1 heterodimer complex
XBP1-d1	XBP1	172 ± 65	≥ 5,000	1.02
JUN-d1	JUN	5.8 ± 0.7	543 (ATF4)	1.07
ATF4-d1	ATF4	9.3 ± 2.3	402 (MAFB)	1.16
ATF5-d1	ATF5	1,680 ± 1,050	657 (CREB3L3)	1.06

To determine the interaction specificity of the bZIP-targeting designs, both N- and C-terminally labeled peptides were tested for binding to 32 different C-terminally fluoresceinated bZIPs at three temperatures (Fig. 3 and S4 Fig.). These proteins were chosen to represent members of all 18 human bZIP families, as discussed by Reinke et al. This combination of tests potentially allows detection of either parallel or anti-parallel coiled-coil interactions between the design and off-target bZIPs, even if FRET is inefficient between dyes located at opposite ends of a coiled coil. Only parallel interactions were considered in the computational design protocol, as the scoring function was trained and benchmarked using parallel interactions and we did not have an equivalent scoring function for antiparallel interactions. Nevertheless, any off-target binding would be undesirable. Fig. 3 reports the tightest off-target interaction for each design at 37°C. All designs bound to the intended bZIP family most tightly, with JUN-d1 showing 100-fold specificity for JUN, ATF4-d1 and XBP1-d1 showing 30-fold specificity for ATF4 and XBP1, and ATF5-d1 showing ~20-fold specificity for ATF4 at 37°C. In all four cases, more than 75% of off-target interactions were not detected or assigned a K_d_ ≥ 5,000 nM at 37°C.

For the five design-target interactions (including interaction of ATF5-d1 with both ATF4 and ATF5), the FRET efficiency was higher when the fluorophores were both located at the peptide C-terminus, rather than one at the N- and one at the C-terminus, supporting a parallel alignment of helices (S3 Table). For some off-target interactions, the FRET efficiency for interaction with the N-terminally labeled design was greater than that with the C-terminally labeled design, suggesting a possible anti-parallel interaction that was not considered in computational design. For example, the FRET efficiency of the interaction between ATF5-d1 and BATF2 was larger when the designed peptide was labeled at the N-terminus than when it was labeled at the C-terminus (0.65 vs. 0.31, respectively, at 4°C). Analysis of potential **e**-**g*'*** charged-residue interactions in the sequences of these proteins indicated one net repulsive interhelical salt bridge in a model of a parallel heterodimer, and four net favorable salt bridges in a model for an antiparallel structure. Similar arguments apply to 5 of the 12 examples where FRET efficiencies were consistent with an anti-parallel helix alignment. Electrostatic interactions between residues at **e** and **g** positions are known to be important for determining helix orientation specificity [36,37].

For ATF4-d1, which forms a strong homodimer (K_d_ of 36.3 nM at 23°C) we repeated 18 of the binding experiments by titrating FRET acceptor-labeled native bZIPs into donor-labeled designed peptide, rather than the opposite [18]. This allowed us to keep the design fixed at a low concentration, potentially making it easier to detect complexes that compete with design homodimer formation. These experiments did not reveal any new interactions except that, for four human bZIPs for which binding to ATF4-d1 was previously weakly detected (CREBZF, BATF, ATF3 and DBP), a complex signal corresponding to increasing donor fluorescence was observed at 4°C. This signal could not be fit well with our binding model, suggesting that a different orientation or oligomerization state might be forming at low temperatures. There was no evidence for interaction at 23 or 37°C when ATF4-d1 was the donor. For these four human bZIPs, ATF4-d1 was tested in a competition experiment at 37°C, to see if the unlabeled design could inhibit a FRET complex containing the candidate binding partner, but no inhibition was detected (S5 Fig.).

**Fig 4:**
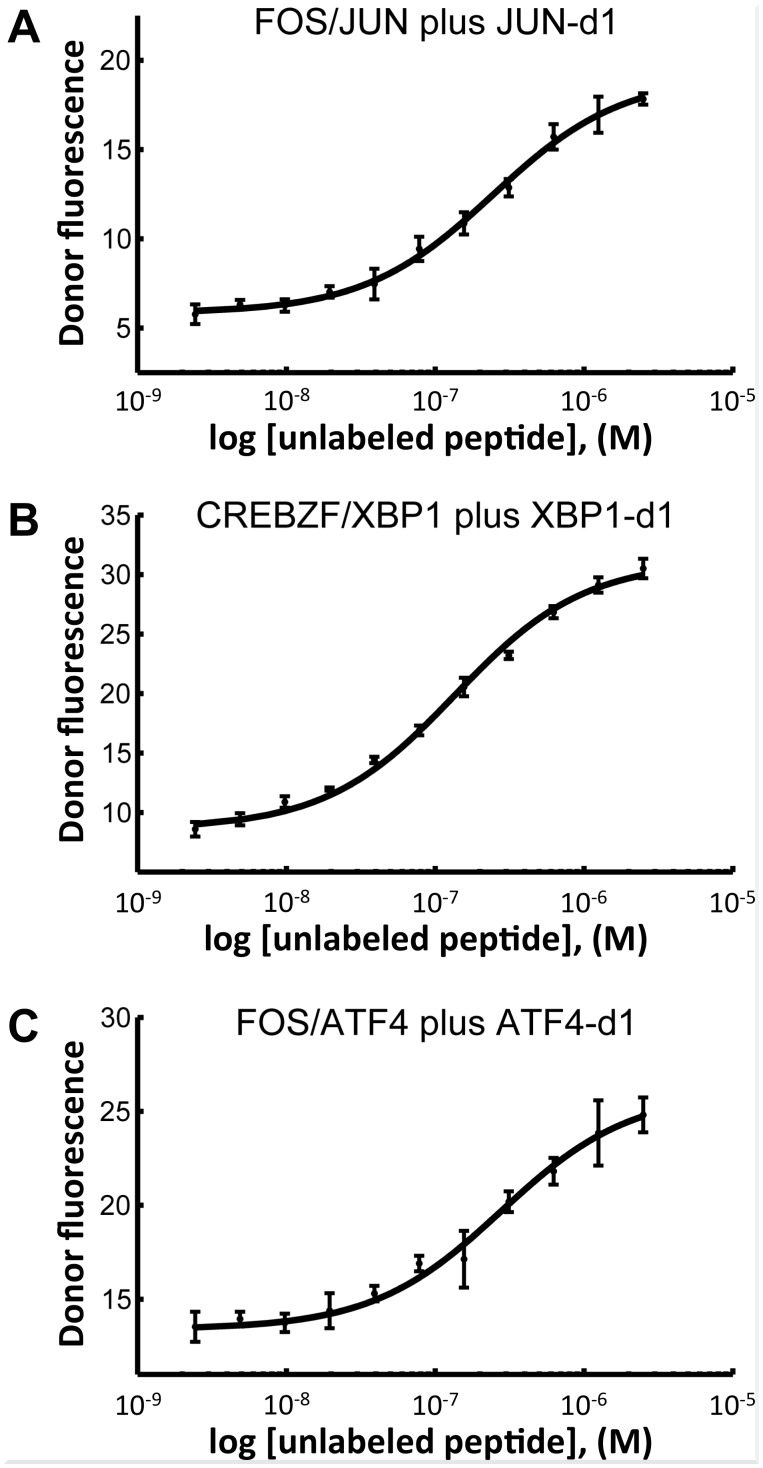
Designed bZIP-binding peptides inhibit interactions of native bZIP dimers. (A) JUN-d1 inhibits the interaction of 10 nM JUN with 50 nM FOS with an IC_50_ of 245 nM at 37°C. (B) XBP1-d1 inhibits the interaction of 10 nM XBP1 with 50 nM CREBZF with an IC_50_ of 136 nM at 23°C. (C) ATF4-d1 inhibits the interaction of 10 nM ATF4 with 200 nM FOS with an IC_50_ of 279 nM at 37°C. The dissociation constants at the indicated temperatures are K_d_ ≤ 1 nM for FOS-JUN, K_d_ ≤1 nM for XBP1-CREBZF, and K_d_ = 60 nM for ATF4-FOS, according to [18]. Fluorescence intensities were measured at both 37°C and 23°C, and the IC_50_ value was fit and reported for the highest temperature that gave a well-defined lower baseline. The target bZIP in each complex was labeled with the FRET donor (fluorescein), the partner was labeled with the TAMRA FRET acceptor, and the design was unlabeled. Fluorescence emission was monitored at 525 nm and is reported in relative fluorescence units.

For most bZIP pairs, binding of the N-terminally labeled designed peptide to a C-terminally labeled target gave very little signal, and such curves could rarely be fit to provide K_d_ values (S9–S12 Tables). In a few cases where interactions could be quantified with both N- and C-terminal dye labeling, we observed that the affinity of N-terminally labeled designs for C-terminally labeled targets was somewhat weaker than the affinity of C-terminally labeled designs (S5–S12 Tables). Enhanced affinities attributable to dye interactions have been reported previously [18]. To assess whether the designed coiled coils could bind tightly to their intended targets without any contribution from the dye, we used the designs in inhibition assays where signal comes from disrupting a FRET complex between two human bZIPs (see Methods). This test mimics applications where designed inhibitors could be used to block bZIP dimerization function. We tested JUN-d1 inhibiting a JUN/FOS heterodimer, XBP1-d1 inhibiting an XBP1/CREBZF heterodimer, and ATF4-d1 inhibiting an ATF4/FOS heterodimer. In all cases, the designs decreased FRET signal from the target complex, with IC_50_ values of ~140–280 nM (Fig. 4). We also demonstrated that unlabeled ATF4-d1 and ATF5-d1 could inhibit complexes between FITC-labeled versions of the designs and TAMRA-labeled ATF4 (K_i_ = 6 nM for ATF5-d1, 48 nM for ATF4-d1; S4 Table). Of note, JUN-d1 was effective at blocking formation of the JUN/FOS heterodimer (also known as the AP1 transcription factor), a functionally relevant complex important for regulating cell proliferation. Mis-regulation of AP1 leads to oncogenic transformation [31]. The dissociation constant of this heterodimer is less than 1 nM [18], and the design inhibits this interaction with an IC_50_ of about 245 nM under the assay conditions.

We analyzed the heptads composing our four most successful designs XBP1-d1, JUN-d1, ATF4-d1 and ATF5-d1. Interestingly, none of the heptads were drawn from native partners of the target proteins, or even from any human proteins (a native partner was defined as any protein from the same species with binding affinity to the target K_d_ < 250 nM). For example, XBP1-d1 was composed of heptads from *N*. *vectensis*, *C*. *elegans* and *C*. *intestinalis* proteins, and from previously designed bZIP binders. We also note that our four designs were composed of 24 distinct, non-recurring heptads. Thus, our design algorithm did not simply re-use heptads coming from native binders, rather it drew diverse modules from a library. We computed all-vs.-all sequence identities for the native and designed proteins in this study (full details are available in Data S1). When considering all seven heptad positions, our four most successful designs, XBP1-d1, JUN-d1, ATF4-d1 and ATF5-d1, have 48%, 45%, 33% and 34% sequence identity, respectively, with a native bZIP coiled coil. When considering the core **a**, **d**, **e**, **g** positions, the same designs have at most 56%, 62%, 42% and 45% sequence identity. Thus, the designs are substantially different from existing proteins. Nevertheless, it is interesting to see some trends. Human ATF6, which binds tightly to XBP1 (K_d_ = 1 nM), has the highest similarity (56%) with our XBP1-d1 design [18], and human FOS, which binds tightly to JUN (K_d_ = 1 nM), is the most similar to our JUN-d1 design (62%). However, ATF6B, the most similar human protein to ATF4-d1 (42%), does not interact with ATF4 (K_d_ ≥ 5,000 nM). ATF5-d1 had a maximum of 38% sequence identity to any human bZIP. Out of five proteins that shared this degree of sequence identity, only CEBPG binds to ATF5 tightly.

Even though some designs share sequence features with human proteins that bind the same target, the interaction profiles of the native and designed protein binders are very different. When considering only strong binders with K_d_ < 250 nM at 21°C, ATF6 interacts with XBP1 and ATF6B and also forms a homodimer. In the same conditions, XBP1-d1 bound only its target, XBP1, and did not show detectable homodimerization. FOS binds 11 human bZIPs including JUN, whereas JUN-d1 binds only JUN, its close homolog JUNB, ATF2 and ATF4. At a higher temperature, JUN-d1 interacted detectably only with JUN and JUNB. CEBPG interacts with 13 human proteins including ATF4 and ATF5, whereas ATF5-d1 interacts with ATF4, closely related ATF5 and possibly with ATF6B. Finally, ATF6B binds XBP1 and ATF6 and forms a homodimer, whereas our design ATF4-d1 binds its target and CREB3L1 and forms a homodimer. Therefore, our designed peptides are more selective than native bZIP coiled coils that bind the same targets, consistent with the main objectives of our design protocol.

Knowing that the native bZIP proteins display limited variability at core **a**, **d**, **e** and **g** positions, it is not unexpected that native partners and computational designs share some features. For example, for targets for which the **d** positions are predominantly occupied by Leu, the tightest binders will require a matching Leu at their **d** positions. There is an even stronger tendency to pair Asn residues with other Asn residues, or sometimes Lys residues, at **a** positions. Thus, XBP1 with three Asn residues at **a** positions will require three matching Asn or Lys residues for any tight binder. Despite some basic requirements for tight binders, there remain many ways to achieve tight and specific binding.

## Discussion

Understanding the detailed relationship between protein sequences and their interactions is an outstanding challenge in protein science. So far, predicting these relationships accurately using purely computational models has not been practical for most systems. Combining computational methods with experiment offers a promising route forward [38,39]. In this work, we started with a large set of experimental data, built a model that can be used in prediction and design, and then applied the model to four instances of a challenging multistate design problem, demonstrating that the model has utility for evaluating new sequences not previously sampled experimentally.

The bZIP coiled-coil interactions have been extensively studied, and it has been demonstrated that pairs of residues at specific positions play defining roles encoding interaction specificity in this family of proteins [6,15,17,29]. The small set of 62 **a**_**i**_-**a*'***_**i**_ and **g**_**i**_-**e*'***_**i+1**_ coupling energies measured in the Vinson laboratory provides a surprisingly successful approach to distinguish strong binders from weak/non-binders [6,17,22] (Table 1). One way to further improve predictions might be to measure more coupling energies, or to make measurements that can account for the context dependence of residue-pair contributions [40]. An alternative is to measure interactions between a large number of bZIP proteins and then computationally deduce the contributions of subsets of residues to overall stability [1,14]. Although earlier peptide array studies provided an overview of human bZIP dimerization preferences [1], the recent work of Reinke et al. dramatically expanded the quantity and resolution of interaction data available for this system [18]. Using reported interaction affinities for more than 4,500 bZIP pairs, we applied a machine-learning approach to computationally derive pair and triplet “coupling energies”. Our computationally derived weights showed excellent agreement with a subset of experimentally measured coupling energies (Figs. 2A and 2B). The linear model is easily interpretable and provides testable hypotheses regarding the contributions of different residue interactions to bZIP stability (e.g. Figs. 2C and 2D).

Our model derived from experimental data was significantly more accurate than other available interaction prediction methods (Tables 1 and 2) and we tested its validity by designing specific binders against four bZIP targets: XBP-1, JUN, ATF4 and ATF5. Because our model is data-derived, it learned only about features (pair and triplet residue interactions) that were observed in the experimental dataset used to build the model. Therefore, in our design strategy, we decided to work in a sequence space similar to that of native bZIP proteins. We devised a modular approach in which elementary repeating building blocks, heptads, were extracted from known bZIP proteins and assembled into new bZIP binders. Using heptads from real proteins allowed us to circumvent the necessity of finding suitable residue candidates for surface-exposed **b**, **c** and **f** positions, as required in previous work by Grigoryan et al. [2]. Overall, our strategy was remarkably successful in scoring and designing specific bZIP coiled-coil interactions (Table 3 and Fig. 3).

We compared the new designs to designed peptides targeting XBP1 and JUN reported by Grigoryan et al. for which FRET measurements were available [25,41]. The previous design for XBP1 showed no detectable binding (K_d_ ≥ 5,000 nM at 37°C), and a previous design for JUN interacted weakly and non-specifically (K_d_ of 482 nM at 37°C). In contrast, the new design strategy coupled with a data-driven scoring function resulted in strong and specific binders. It should be noted that XBP1 presents a challenging target because three of its core **a** positions are occupied by asparagines. A matching asparagine is strongly favored at the opposing **a** positions by the design algorithm (consistent with preferences in native proteins), but Asn-Asn^aa*'*^ is destabilizing relative to a pair of hydrophobic residues at these positions [6,23]. Nevertheless, our combined approach led to a successful XBP1 binder (XBP1-d1). Our second XBP1 design, XBP1-d2, did not bind detectably to XBP1. However, mutation of two residues to the corresponding residues in XBP-d1 restored binding. One of these mutations was a change from a tyrosine to a leucine at an **a** position. Noting that tyrosine residues are rarely observed at the **a** positions of known bZIPs, it is likely that weights for pairs and triplets including tyrosine, or other rare residues, were poorly determined. This reasoning contributed to our decision to limit the choices of amino acids at the **a**, **d**, **e** and **g** positions to commonly occurring residues.

In addition to XBP1-d2, there were a few other designs that were predicted to bind tightly to their targets but that did not (Table 3). In particular, none of the designs targeting ATF3 were strong binders. Also, ATF5 designs exhibited significant variability: ATF5-d1 was predicted to bind both ATF5 and ATF4 (with a better score for ATF4 than ATF5), but bound strongly to ATF4 only. There is clearly room for improvement in the scoring function, which approximates interactions between two or three residues with a single weight independent of structural context. As was seen for XBP1-d2, sometimes small changes in protein sequence have a dramatic effect on binding, and our model does not always capture that. Interactions that are infrequent in the training set present a particular challenge, and strategically increasing the diversity of the training data might make it possible to derive more reliable weights. Another way to improve the design success rate could be to include additional constraints disfavoring off-target interactions. For example, we noted that some of our designs bound off-targets in an anti-parallel arrangement. The model described in this study was developed for predicting parallel bZIP coiled-coil interactions only, and could not be used to score anti-parallel alignments. However, recently developed models for anti-parallel coiled coils could be included in future designs using our approach [42,43]. Additional constraints could also be included to account for net charge, helix stability and/or aggregation propensity [44,45]. ILP is optimally suited for incorporating constraints while optimizing the binding affinity of designed sequences.

During the computational optimization of designed sequences we imposed certain knowledge-based constraints on amino-acid composition. These constraints had two effects. First, the constraints reduced the complexity of the optimization problem. Limiting the selection of heptads to those containing the most common amino acids at **a**, **d**, **e** and **g** positions (as discussed below and detailed in Methods) was important for computational tractability and ILP convergence. Second, the constraints forced the designs into a sequence space that more closely resembled the amino-acid composition of native bZIPs on which our scoring function was trained. For example, constraints imposed a limit on the number of polar residues in core positions, and restricted residues like His to heptads in specific locations in the designed sequence (see Methods for details). Designing binders under sequence-composition constraints typically decreased the predicted binding affinity (K_d_) by ~3 fold, compared to designing without constraints. However, even with the constraints, the predicted scores were in a range where we still expected tight binding to the target. Constraints on amino-acid composition were usually introduced after examining designed sequences and identifying certain features that we wanted to avoid based on prior knowledge. For example, ATF5 designs tended to have several methionine residues in core **d** positions. Although Met at **d** is common, it is not common to see more than one Met per sequence in native bZIPs. Similarly, some ATF4 designs had more polar residues in core **a** positions than are observed in native bZIPs. Polar residues are common at **a** positions and are required to encode interaction specificity, but these groups are destabilizing relative to hydrophobic residues, and we tried to avoid excessively polar sequences. Interestingly, successful design JUN-d1 violated one of our compositional constraints (this sequence came from a design calculation with slightly different constraints that allowed glutamate in non-terminal heptads), demonstrating that these restrictions are not essential. Nevertheless, we recommend them as part of a conservative strategy focused on finding solutions with native-like composition.

The heptad library from which we assembled designed sequences was composed of 1,303 unique heptads, when considering amino-acid residues in all seven positions; when considering only **a**, **d**, **e** and **g** positions, there were 660 heptads with distinct sequences to choose from. As mentioned in the Methods, the **a** position in 85% of bZIP sequences is occupied by one of the 8 residues Leu, Asn, Val, Ile, Lys, Ala, Arg or Thr, and the **d** position is occupied by one of the 5 residues Leu, His, Val, Met or Ile. Similarly, positions **g** and **e** are predominantly occupied by Glu, Lys, Arg, Gln or Leu. Thus, 1,000 heptads (8 x 5 x 5 x 5) can form a fairly complete basis set covering **a**, **d**, **e** and **g** positions. Our heptad library covered only 66% of this space, and it is worth considering whether the library should be supplemented with the “missing” residue combinations. Without some special motivation to expand the search space (such as many failed designs), we feel that there are advantages to working with the more restricted set of known heptads. These heptads have survived natural selection, or have been demonstrated to form coiled coils in prior design work, and may account for residue-pair dependencies within a protein chain that are ignored in our model. For example, native heptads provide **b**, **c** and **f** position residues that are compatible with **a**, **d**, **e** and **g** residue choices. Native heptads may also result in better protein solubility.

In conclusion, our work provides a state-of-the-art method for predicting and designing bZIP-like peptides for selectively inhibiting transcription factor dimerization. Short, designed coiled coils also have potential applications in systems biology, and in constructing novel sequences that can assemble into nanoscale materials [46–48]. Ours is an approach in which experimental data, coupled with computational learning techniques, provided a model that helped us learn more about a protein-interaction system and engineer new functions. This sort of approach will likely be useful for addressing a wider range of protein interaction problems in the future, as more experimental data become available.

## Methods

### Experimental data used for model training and testing

A large set of bZIP coiled-coil interactions quantified by Reinke et al. provided the data for model building and testing [18]. In the Reinke et al. work, if a K_d_ value was determined to be >5,000 nM, or no binding was experimentally detected, then a K_d_ value of 5,000 nM was assigned. Each interaction was measured twice, switching the identity of the donor vs. acceptor labeled peptides in FRET titrations; the lower of the two K_d_ values obtained was used. In total, 5,271 bZIP coiled-coil interactions were quantified experimentally.

We curated the data to remove interactions for which we were unsure of the alignment of sequence to structure, which can be inferred with confidence for most bZIPs based on known structures plus a multiple-sequence alignment constructed from the DNA-binding and coiled-coil regions [1,29]. First, a number of bZIP coiled-coil pairs in the data set have asparagine at one or more **a** positions in one chain and valine, leucine, or isoleucine at the opposing **a** position in the second chain, according to sequence alignment. These so-called asparagine mismatches are known to strongly destabilize bZIP complexes [6], and such pairs may not be forming dimers with the canonical helix alignment inferred from sequence alignment; thus, such examples were excluded from the dataset. In total, 120 interactions with asparagine mismatches in non-terminal heptads of the coiled-coil motif were excluded. Second, some bZIP proteins had numerous uncharacteristic residues at the core **a**, **d**, **e** and **g** positions that made the alignment between chains in a dimer uncertain. To identify such proteins systematically, a position-specific scoring matrix (PSSM) for residues at **a**, **d**, **e** and **g** positions was computed based on sequences of proteins in our experimental bZIP dataset. The PSSM scores were defined as $s_{i}^{aa}=-log\frac{p_{i}(aa)}{p(aa)}$, where i is the heptad position (**a**, **d**, **e** or **g**), aa is the amino acid residue, *p*_*i*_(*aa*) is the probability to observe amino acid residue aa at position i, p(aa) is the probability to observe amino acid residue aa in any position. Amino-acid residues that frequently occupy a given position accordingly get a low score $s_{i}^{aa}$. All sequences were scored using PSSM scores, and 10% of proteins with the highest scores (22 in total), and all interactions in which these proteins participated (555 in total), were excluded from the dataset. Finally, the dataset contained 56 pairs of interactions formed by different length variants of the same proteins. Such interactions had different K_d_ values but identical coiled-coil regions, despite different chain lengths for the experimental constructs. These cases were also excluded from the dataset because they represented conflicting K_d_ values for the same interaction motif. The final dataset contained 4,549 interactions; among them were 948 binders (K_d_ < 5,000 nM) and 3,601 weak/non-binders (K_d_ ≥ 5,000 nM). The logarithms of experimentally determined K_d_ values, log_10_(K_d_), were used throughout this study. The interactions involved 200 bZIP proteins from 8 species: *H. sapiens* (45), *C. elegans* (25), *C. intestinalis* (34), *N. vectensis* (37), *D. melanogaster* (27), *M. brevicollis* (19), *S. cerevisiae* (11) and *D. rerio* (2). Interactions were measured between proteins from the same species (intra-species interactions) and between proteins from different species (inter-species interactions). Additionally, some interactions were measured between different variants of the same proteins (mutants and different-length variants) and other wild-type and variant proteins. There were 2,302 intra-species interactions, 1,134 inter-species interactions and 1,113 interactions among variants. The dataset used for our analyses is available in Data S2.

The reproducibility of the experimental data was evaluated in two ways. As mentioned above, each bZIP interaction was measured twice. All interactions were identified for which at least one of the two K_d_ values was less than 5,000 nM and neither of the interacting proteins formed a homodimer (homodimer K_d_ ≥ 5,000 nM). The Pearson correlation coefficient for this set of K_d_ values was R = 0.86 for 103 pair measurements. In the second test, the Pearson correlation coefficient was computed for a set of 280 intra-species interactions (173 from *H. sapiens* and 107 from *C. elegans*) with two alternative measurements each, which came from two alternative titration procedures in the FRET experiments (one manual, one done by a liquid-handling robot). The resulting correlation coefficient R was 0.82.

### Feature encoding and weight optimization

The model was based on the assumption that interactions between residues in the coiled-coil interface are the major contributors to bZIP interaction specificity, as is supported by earlier work [5,6]. Therefore, each bZIP dimer was represented as a set of discrete pair and triplet residue interactions (as defined below), and the optimization framework was applied to deduce the contributions of individual interactions to the overall stability of the bZIP dimer. Pairs were defined between residues from opposing chains at **a**, **d**, **e** and **g** positions that are close in structure and can potentially form a contact in the coiled-coil motif. In total, eight distinct types of pairs were considered: **a**_i_-**a*'***_i_, **d**_i_-**d*'***_i_, **g**_i_-**e*'***_i+1_, **a**_i_-**d*'***_i_, **d**_i_-**a*'***_i+1_, **d**_i_-**e*'***_i_, **g**_i_-**a*'***_i+1_ and **e**_i_-**g*'***_i_, where the letter indicates structural position in the coiled-coil motif, the index indicates a heptad and the primes indicate an alternate chain [14]. Triplets were defined as sets of three residues at **a**, **d**, **e** and **g** positions in the coiled-coil interface for which at least two potential contacts could be formed among residues in the structure. Ten types of triplets were considered: **a**_i_-**d**_i_-**a*'***_i_, **d**_i_-**a**_i+1_-**d*'***_i_, **g**_i_-**d**_i+1_-**e*'***_i+1_, **g**_i_-**a*'***_i+1_-**e*'***_i+1_, **e**_i_-**d*'***_i_-**g*'***_i_, **e**_i_-**a**_i+1_-**g*'***_i_, **a**_i_-**g*'***_i-1_-**d*'***_i_, **a**_i_-**e**_i_-**d*'***_i_, **d**_i_-**e*'***_i_-**a*'***_i+1_, **d**_i_-**g**_i_-**a*'***_i+1_. Combinations of residues were denoted in the following way: for pairs, Asn-Asn^aa**`**^, and for triplets Leu-Asn-Leu^dad*'*^, where the type of pair or triplet is given by a superscript. For every type of pair, there could be up 400 individual interactions, and for every type of triplet there could up to 8,000 individual interactions, because each position in a pair or a triplet can be occupied by any one of the 20 amino acids. The two pairs **a**_i_-**a*'***_i_ and **d**_i_-**d*'***_i_ are symmetric with respect to which residue is in which chain. Therefore, there are only 210 individual interactions for these pairs. The pairs **g**_i_-**e*'***_i+1_, **e**_i_-**g*'***_i_, were also treated as symmetric (no distinction was made between which residue was in which chain), because interaction energies between long-chain residues that are common and **e** and **g** sites (Glu, Gln, Lys, Arg) are similar for reversed pairs [5]. It should be noted that although the total number of features in our model is 82,440, only a small fraction of the possible residue-residue interactions are represented in our dataset of 4,549 bZIP interactions: 1,930 individual pair interactions and 17,239 individual triplet interactions were observed at least once in our dataset.

To formulate model building as an optimization problem, we assumed that each individual interaction, referred to here as a feature, contributes independently to the coiled-coil stability (*E*). The overall stability is expressed as a sum of contributions: $E=\sum_{i=1}^{N}w_{i}x_{i}$, where *x*_*i*_ is the number of times the *i* ^*th*^ feature is seen in the coiled coil, *w*_*i*_ is the contribution of the *i* ^*th*^ feature to coiled-coil stability, and *N* is the total number of features. Semi-quantitative support vector regression, similar to the method of [21], was used to optimize a linear additive model, defined above, under two types of constraints: $\left|E_{k}-E_{k}^{\exp}\right|\leq \varepsilon +\xi_{k}^{1}$ for binders (1 nM < K_d_ < 5,000 nM) and $E_{k}\geq E_{cutoff}+\xi_{k}^{2}$ for weak/non-binders (K_d_ ≥ 5,000 nM), where *E*_*k*_ is the predicted coiled-coil stability, $E_{k}^{\exp}$ is the experimentally measured stability, *E*_*cutoff*_ is a predefined non-binder cutoff value, *ε* is a tolerance threshold, and $\xi_{k}^{1}$ and $\xi_{k}^{2}$ are slack variables allowing violation of constraints for binders and non-binders, respectively. Subject to the above constraints, we minimized the objective function ${\left|w\right|}^{2}+C_{1}\sum_{k=1}^{m}\xi_{k}^{1}+C_{2}\sum_{k=1}^{n}\xi_{k}^{2}$, where |*w*| is the norm of the weight vector, *m* and *n* are the number of binders and non-binders, respectively, and *C*_*1*_ and *C*_*2*_ are predefined constants. IBM ILOG CPLEX Optimizer was used to solve the optimization problem [49]. The parameters *C*_*1*_, *C*_*2*_ and *ε*, which affect the performance of the resulting model, were identified through grid search using cross-validation, as described below.

### Cross-validation

To estimate the generalization error of our machine-learning model and identify optimal values of parameters *C*_*1*_, *C*_*2*_ and *ε*, we used nested cross-validation. The first step of the procedure was to divide the entire experimental dataset *S* into 10 non-overlapping and minimally similar partitions *S*_*i*_,_*i = 1*..*10*_. The model was trained ten times, each time holding out one of the partitions *S*_*i*_, training the model on the remaining dataset *T* = *S* \ *S*_*i*_, and then evaluating the obtained model on the held-out set *S*_*i*_. The cross-validated performance was estimated by recombining the results of individual evaluations on the held-out partitions *S*_*i*_ and computing the performance metrics (R and AUC). Importantly, training the model required choosing optimal values of *C*_*1*_, *C*_*2*_ and *ε* parameters, which in turn involved cross-validation. Therefore, at each above-described training step, the dataset *T* was in turn divided into 10 partitions *T*_*k*,*k = 1*..*10*_. Optimal values for *C*_*1*_, *C*_*2*_ and *ε* were identified by trying different combinations, using grid search, and choosing a combination that had the best cross-validation results obtained on *T*_*k*_ datasets. As a result, model training (including choosing the parameters) and evaluation were always performed on separate datasets, thus allowing for accurate estimation of model generalizability. Note that applying nested cross-validation resulted in ten potentially different optimal choices of *C*_*1*_, *C*_*2*_ and *ε* parameters, one for each *S* \ *S*_*i*_. These were used to estimate the generalized performance. The final model was obtained by taking the combination of *C*_*1*_, *C*_*2*_ and *ε* parameters used for the *S*_*i*_ test set that gave the best performance. The final model was used to design specific bZIP binders.

Using cross-validation requires dividing a dataset into several partitions of equal size with sufficiently different training examples. We applied a graph-theoretical algorithm to partition the training set. Because each coiled coil was represented as a feature vector in this study, we first computed Jaccard distances between all coiled coils in the dataset. The Jaccard distance is the percentage of non-zero features that differ between two coiled coils. Then, the graph-partitioning tool METIS was used to divide the training set into partitions of equal size with minimal overlap between partitions [50]. Additionally, we required that coiled coils of different binding strengths were equally represented in the resulting partitions to avoid a bias: each partition had ~80% non-binders (K_d_ ≥ 5,000 nM), 10% strong binders (K_d_ < 50 nM) and 10% weaker binders (50 nM < K_d_ < 5,000 nM). The resulting partitions are available in Data S2. The experimental dataset was additionally divided into training and test sets for certain evaluations. This was achieved by partitioning the entire set into ten non-overlapping and minimally similar subsets, as described above, and combining the first seven partitions into the training set and remaining 3 partitions into the test set.

To evaluate how the size of the experimental dataset influenced model performance, a series of reduced datasets containing from 10% to 100% of the total interactions was generated. Then model optimization and nested cross-validation were repeated as described above on each of the reduced datasets. The results are presented in S1 Fig.

### Feature selection

Recursive feature elimination (RFE) was used to identify a reduced subset of features sufficient for accurate prediction of bZIP interaction specificity [51]. Following the selection of *C*_*1*_, *C*_*2*_ and *ε* parameters in the inner loop of the nested cross-validation, the full feature vector was subjected to RFE with the chosen *C*_*1*_, *C*_*2*_ and *ε* parameters. This involved three iterative steps: (1) all features were ranked according to the absolute value of their corresponding feature weight, (2) 5% of features with the lowest weights were discarded, (3) the remaining features were retrained. This procedure was repeated until no features were left in the feature vector, tracking model performance at each step. The reduced feature vector with the best performance was identified and subsequently utilized in the outer loop of the cross-validation, in conjunction with the optimal *C*_*1*_, *C*_*2*_ and *ε* parameters, to estimate the generalization error of a model.

### Benchmarking

A number of methods were used to calculate stability scores for bZIP coiled-coil interactions in our dataset. The data-derived methods rely on scoring interactions between residues in different positions in the coiled-coil motif. The method of Fong et al. (referred as Fong/SVM in the text) was developed to distinguish between coiled-coil binders and non-binders [14]. The authors derived a set of 1,325 computationally optimized weights accounting for pairs of residues in **a**_i_-**a*'***_i_, **d**_i_-**d*'***_i_, **g**_i_-**e*'***_i+1_, **a**_i_-**d*'***_i_, **d**_i_-**a*'***_i+1_, **d**_i_-**e*'***_i_ and **g**_i_-**a*'***_i+1_ positions. We applied this set of weights to score coiled-coil interactions in our benchmarks. Similarly, we used a set of 61 coupling energies determined experimentally for most commonly occurring residues in **a**_i_-**a*'***_i_ and **g**_i_-**e*'***_i+1_ positions by Vinson and colleagues (referred as Vinson/CE in the text) [5,6]. We added an extra -2 kcal/mol weight for Leu-Leu in **d**_**i**_-**d*'***_i_ position, as this interaction is known to strongly stabilize coiled-coils and improve the performance of scoring using this approach [16]. We also used the bCIPA algorithm as provided by the web server at http://www.molbiotech.uni-freiburg.de/bCIPA/, which was parameterized to predict coiled-coil melting temperatures from sequences, guided in part by the coupling energies used in model Vinson/CE [15].

Structure-based predictors required prediction of structure from sequences. For this purpose, the coiled-coil Crick parameterization server was used to generate a backbone template based on the crystal structure of the coiled coil from yeast bZIP GCN4 (PDB ID: 2zta), as was done in previous work [16]. For HP/S/C scoring, we generated structural models by placing side chains using the CHARMM19 force field, the Richardson penultimate rotamer library and dead-end elimination, as described in [16]. Only **a**, **d**, **e** and **g** heptad positions were considered; **b**, **c** and **f** were fixed as Ala. The resulting models were scored using HP/S/C [16]. For Rosetta scoring, we used the fixed-backbone application to pack side chains at **a**, **d**, **e** and **g** positions on the backbone template; **b**, **c** and **f** position were fixed to Ala (modeling residues at all heptad positions had a negligible but negative effect on the final result). One hundred structures were generated for each coiled coil using the Rosetta soft potential to compensate for use of a fixed backbone (*ffixbb.linuxgccrelease -packing:ex1 -packing:ex2 -packing:repack_only -packing:extrachi_cutoff 1 -correct -minimize_sidechains*). A structure with the lowest score was retained. Then, the complex and individual subunits, obtained by deleting the opposite chain, were scored and the final score was computed as *E*_*Complex*_ *—E*_*Chain A*_ *—E*_*Chain B*_. Repacking side chains in isolated subunits did not result in improved overall accuracy in our benchmarks. Alternatively, the default Rosetta potential ‘*score12*’ was used to pack side chains in the dimer (*fixbb.linuxgccrelease -packing:ex1 -packing:ex2 -packing:repack_only -packing:extrachi_cutoff 1 -correct -minimize_sidechains*) followed by relaxation of the structure using ‘*relax*’ (*relax.linuxgccrelease -in:file:fullatom -relax:thorough -packing:ex1 -packing:ex2 -packing:repack_only -packing:extrachi_cutoff 1 -correct -minimize_sidechains*). Modeling with the soft Rosetta potential gave better results, which were reported. For dDFIRE scoring, we used the distance-dependent all-atom potential dDFIRE to score HP/S/C and Rosetta-generated structures [19]. The best result was achieved when the final score was computed as *E*_*Complex*_ *—E*_*ChainA*_ *—E*_*ChainB*_ with a distance cutoff of 8 Å. Scoring both HP/S/C- and Rosetta-generated structure gave similar results, and only results obtained on the latter were reported.

### Design procedure

In our design procedure, we aimed to design peptides that would bind tightly to only the defined target while making minimal interactions with other bZIP proteins. Fifty-one human bZIP proteins were considered in the calculations [1]. bZIP proteins belonging to the same protein family as the target protein were not considered as off-targets because there is little sequence difference between family siblings.

In our approach, seven-residue sequence fragments referred to as heptads were used to assemble new sequences, and integer linear programming was used to find the optimal combinations of heptads to make a strong and specific binder. To achieve this goal, a library of heptads was constructed by extracting seven-residue fragments from 340 bZIP sequences, including sequences from the current experimental dataset, other human bZIPs [1], previously designed bZIP binders [2] and zebrafish bZIPs (personal communication, A.W. Reinke). The first residue of each heptad corresponded to an **f** position. There were 1,303 unique members in the heptad library (*N*). The target sequences were also cut into *M* heptads starting at **f** positions, and interaction scores $s_{i}^{j}$ between heptads in the library and heptads in target bZIP proteins were pre-computed; *j* is the index of a heptad in the library (*j = 1*..*N*) and *i* is the index of a heptad in the target protein (*i = 1*..*M*). We additionally computed correction scores $c_{i,i+1}^{j,k}$ to account for differences in scores $s_{i,i+1}^{j,k}$ when two consecutive library heptads *j* and *k* interact with two consecutive *i* and *i+1* heptads in the target protein. These additional terms arise from triplet terms in the scoring function. With all elementary interactions pre-computed, the overall score for any target protein can be expressed as follows:

$$S=\sum_{i}^{M}\sum_{j}^{N}x_{i}^{j}s_{i}^{j}+\sum_{i}^{M-1}\sum_{j}^{N}\sum_{k}^{N}z_{i,i+1}^{j,k}c_{i,i+1}^{j,k},$$

where $x_{i}^{j}$ is a binary decision variable indicating whether heptad *j* in position *i* is part of the optimal solution and $z_{i,i+1}^{j,k}$ is another binary decision variable that indicates what correction scores should be applied. Design was done by minimizing the score of a designed binder with its target, *S* ^target^, such that all off-target interactions scores were above a pre-defined cutoff $S_{i}^{\text{off}-\text{target}}>C$ for every off-target *i*. The cutoff value *C* was chosen such that the difference between *S* ^target^ and $S_{i}^{\text{off}-\text{target}}$ was at least greater than two, which corresponded to a >100-fold difference in predicted K_d_ values. Two sets of constraints were imposed to ensure a self-consistent optimal solution: $\sum_{j}^{N}x_{i}^{j}=1$ for all *i*, and also $\sum_{j}^{N}z_{i,i+1}^{j,k}=x_{i}^{k}$ and $\sum_{k}^{N}z_{i,i+1}^{j,k}=x_{i}^{j}$, for all *i*, which ensured that only a single heptad was permitted for each position in the target protein. Additional constraints were added to allow adjacent heptads only when the designed pairs of residues **e**-**f**, **d**-**g** and **e**-**a** within the same chain at the junction of two heptads were observed at least once in natural bZIP sequences. The optimal solution was found using the IBM ILOG CPLEX optimization tool.

We also incorporated a number of knowledge-based rules into our optimization procedure to avoid designing sequences with undesired features. We analyzed the occurrence of different amino acids in bZIP proteins and found that in 85% of heptads the **a** position is occupied by Leu, Asn, Val, Ile, Lys, Ala, Arg or Thr, and the **d** position is occupied by Leu, His, Val, Met or Ile. Thus, only heptads composed of such residues were used in design (1,054 heptads). N-terminal heptads in native bZIP proteins (149 in total) were found to be more polar than heptads from other regions, and based on this difference these heptads were restricted to the most N-terminal position in designed sequences. Other heptads from native proteins were permitted at any coiled-coil position. Asparagine residues in **a** positions are disfavored across from hydrophobic residues [6], and this is an important specificity element in bZIP sequences that is recognized by our scoring function. However, Asn across from a hydrophobic residue appears to be much less unfavorable at the ends of coiled coils [7]. Therefore, heptads with Asn residues at the **a** position were restricted to non-terminal heptads in designs. Heptads having destabilizing **a**_**i**_-**a*'***_**i**_ and **d**_**i**_-**d*'***_**i**_ pairs with the target, with coupling energies > 1 kcal/mol as determined by Acharya et al. [6]), or heptads having rarely observed **a**_**i**_-**a*'***_**i**_ and **d**_**i**_-**d*'***_**i**_ pairs (seen less than twice in strong binders, K_d_ < 250 nM) were not permitted in the designs.

We additionally analyzed the amino-acid composition of bZIP sequences and found distinct patterns that were incorporated into the design algorithm. In native bZIP sequences that we analyzed, His in a **d** position was found in the last (typically 6^th^) heptad in 96% of occurrences; Glu in the **a** position was found in the first heptad in 91% of occurrences; the total number of Met residues in the **d** position in 95% of cases was ≤ 1; the total number of polar residues (Asn, Lys, Arg, Glu, Thr, Ala) in the **a** positions in 95% of the cases was ≤ 4. These rules were added as additional constraints on the optimization procedure.

### Cloning, purification, and labeling of designs and targets

Artificial genes encoding the designed sequences were constructed by synthesis using codon-optimized primers generated by DNAworks [52] and ordered from Integrated DNA Technology. Target leucine-zipper constructs for the AUC analysis were subcloned from previously described constructs [53]. DNA oligonucleotides were digested using BamHI and XhoI (NEB) and ligated into a modified pDest vector containing an N-terminal 6-histidine tag followed by the linker GESKEYKKGSGS to help with solubility [53]. An additional GC or CG was added to the C-terminus or N-terminus (between the solubility linker and design sequence), respectively, for labeling. All clones were sequence verified before expression. Designs were expressed in *E*. *coli* RP3098 cells by growing in 1 L of LB at 37°C to an OD at 600 nm between 0.4–0.6. Protein expression was induced by the addition of 1 mM IPTG and cells were grown for an additional 4 hours before being pelleted. Proteins were purified on a Ni-NTA column under denaturing conditions (6M guanidinium hydrochloride, 20 mM Tris pH 8.0, 0.5 M NaCl, 1 mM DTT) followed by reverse-phase HPLC using a linear acetonitrile gradient [53], after which they were lyophilized and sent for MALDI analysis. All masses were correct to within 1%.

Labeling with fluorescein-5-maleimide or Rhodamine Red C2-maleimide (Invitrogen Life Technologies) was done as described [46]. Briefly, protein was reduced in 1 mM TCEP-HCl (Pierce Technology), buffer-exchanged into degassed PBS (137 mM NaCl, 2.7 mM KCl, 10 mM Na_2_HPO_4_, 2 mM KH_2_PO_4_), and incubated overnight at room temperature with 10-fold excess fluorophore. After labeling, free dye was removed using a Ni-NTA column. Labeled proteins were then lyophilized, re-suspended, desalted using a spin-column (Bio-Rad), and stored in 10 mM potassium phosphate buffer, pH 4.5, at -80°C. Concentrations were measured in 6 M guanidine-HCl/100 mM sodium phosphate pH 7.4 using the absorbance of the dye with an extinction coefficient of 68,000 M^-1^ cm^-1^ at 499 nm for fluorescein and 119,000 M^-1^ cm^-1^ at 560 nm for rhodamine [18,46]. These concentrations were within 2-fold of protein concentrations determined using the extinction coefficient of the peptide at 280 nm and taking into account contributions from the dye. Fluorescein- and TAMRA-labeled target bZIP proteins have been previously described and were generously donated by A.W. Reinke [18].

### Direct FRET assay and competition FRET assay

The direct binding assay was performed manually in 384-well plates and is similar to a previously described assay [18]. Wells were filled with 20 μL of 1 mM TCEP-HCl. A 4 μM stock of acceptor protein was made fresh in 1 mM TCEP-HCl, and 20 μL was titrated in 2-fold dilutions over the wells. Twenty microliters of a 40 nM stock of donor-labeled protein was then added to the wells, and then 40 μL of 2x binding buffer was added to the wells and mixed, for final concentrations of 10 nM donor-labeled protein and acceptor-labeled protein ranging from 0 to 1 μM in a total volume of 80 μL in 150 mM KCl, 50 mM potassium phosphate pH 7.4, 0.1% Tween-20, 0.1% BSA. FRET efficiency was calculated with the equation 1-F/F_0_, where F is the fluorescence value at 1000 nM of acceptor and F_0_ is the fluorescence value at 0 nM acceptor. 260 interactions were tested at three temperatures with the designs as the FRET acceptors. 44 of the binding curves were re-measured at least one time, with a focus on design-target and design-close off-target interactions, and 60 were also tested using the fluorescein-labeled design as the FRET donor and the TAMRA-labeled target protein as the FRET acceptor.

In the competition form of the assay, a FRET pair was made by mixing 10 nM of the donor-labeled peptide with a specified concentration of the acceptor-labeled peptide in 1 mM TCEP-HCl, and 20 μL of this solution was added to the wells after unlabeled design was titrated in 2-fold dilutions over the wells to a final concentration ranging from 0 to 2.5 μM. The amount of acceptor used varied for each curve, taking into account the K_d_ of the FRET complex in order to obtain a good lower baseline for fitting purposes. Samples were equilibrated for one hour before reading the plates; repeated reading of the plates over several hours gave no change in signal.

### Data analysis

The direct-binding FRET data were fit as previously described [18,54]. Briefly, the K_d_ of the designed peptide homodimer was experimentally determined and used with previously determined target homodimer K_d_ values to determine the heterodimer K_d_. The homodimer K_d_ values used for the human bZIPs were from [18]; experiments done by Reinke et al. used the same protein constructs for the human bZIP proteins, the same buffer conditions, and the same assay. The fitting used a system of ordinary differential equations to describe the concentrations of homo and heterodimers formed by the donor (D) and acceptor (A) as a function of the heterodimer K_d_. MATLAB was used to simulate the binding experiment for different K_d_ values, and the value that gave the best agreement between simulation and experiment was assigned. A minimum 15% change in the donor fluorescence upon titrating the acceptor peptide from 0 to 1000 nM was required for a fit to be attempted. If this minimum was not met, the interaction was classified as “NS” for no signal change. All curves that showed a change in signal were fit twice, using an upper limit on the K_d_ of 5,000 nM or 1,000 nM, and fits were individually inspected to ensure quality and proper classification. If the best-fit K_d_ value was 5000 nM, this upper bound K_d_ value was assigned with notation “> 5000”, unless the R^2^ of the fit using 1000 as the upper bound was > 0.95. In this case, the assigned K_d_ was ~1000.

For some peptide pairs, we noted that the fluorescence signal for the first several titration points showed an anomalous increase in signal (often with significant noise), followed by a decrease. These interactions were marked “AS” to indicate anomalous signal. We attributed this to interactions between the peptides, the dyes and the walls of the assay plates at low concentrations. These curves could not be fit, given their unusual shapes at low concentrations of acceptor. For such curves, we removed the first five points and re-fit the data. If the re-fit curve was still very noisy, showing no systematic decrease in donor fluorescence with addition of receptor, the interaction was classified as “NI” for no interaction detected up to 1000 nM of acceptor. For re-fit curves that showed evidence of binding, these were classified as AS - weak (estimated K_d_ > 5000), AS - moderate (estimated K_d_ < 1000), and AS - strong (estimated K_d_ < 200 nM); estimates were made using fits to the truncated curves. Notably, only 6 interactions with anomalous signal appeared consistent with strong binding. Examples of AS curves are included in S6 Fig.

For signal changes that did not fit the model of an acceptor being within range to participate in FRET with a donor, the interaction was classified as “ND” to indicate an interaction was likely occurring but could not be described with the given model. One example of this is increasing concentrations of acceptor causing an increase in donor fluorescence. A candidate model to describe this interaction is the acceptor-labeled design competing with a self-quenching donor homodimer in a geometry that does not permit efficient FRET. Such curves were rare and occurred only for seemingly weak interactions, e.g. in the case of ATF4-d1 with C-terminal fluorescein or interactions that were detectable with the acceptor at the other terminus and were already reported, as in the case of XBP1-d1 with N-terminal rhodamine. S6 Fig. shows examples of ND curves.

For the competition experiments, the IC_50_ was determined in MATLAB by fitting the binding data to the equation $signal=a+\frac{b-a}{{\left(1+x/c\right)}^{H}}$, where *a* and *b* are the lower and upper baselines, *c* is the IC_50_ value, *x* is the inhibitor concentration, and the Hill coefficient *H* was set to 1. K_i_ values were calculated as in Fu et al. [55].

### Analytical ultracentrifugation

Equilibrium AUC was performed using a Beckman XL-I centrifuge with interference optics. Individual proteins were dialyzed three times against a reference buffer (1x PBS + 1 mM TCEP-HCl) including at least once overnight. Concentrations were measured after dialysis, and equal-molar mixtures of the unlabeled design with the leucine-zipper target were mixed to give total peptide concentrations ranging from 40 μM to 200 μM. Three different rotor speeds of 28,000 rpm, 35,000 rpm and 45,000 rpm were run at 20°C for at least 20 hours, and equilibrium was ensured before measurements were taken by checking that the signal did not change between sequential scans. Partial specific volume, buffer density, and buffer viscosity were calculated using the Sednterp web server (Biomolecular Interaction Technologies Center). Data were analyzed using Sedfit [56]. Each concentration was fit individually using data from all three spin speeds. The best-fit molecular weight was calculated for a single species and reported for the sample at 40 µM total peptide.

## Supporting Information

S1 Fig Model performance as a function of the amount of training data. (PDF)

S2 Fig Differential heat maps of the 20 x 20 amino-acid interactions for a_i_-a′_i_, d_i_-d′_i_, g_i_-e′_i+1_ pairs. (PDF)

S3 Fig Analytical ultracentrifugation data for JUN-d1 mixed with JUN. (PDF)

S4 Fig Specificity profiles of designed peptides XBP-d1, JUN-d1, ATF4-d1 and ATF5-d1. (PDF)

S5 Fig ATF4-d1 does not inhibit FRET complexes formed by CREBZF, DBP, ATF2 or BATF bZIP coiled coils. (PDF)

S6 Fig Representative plots of the different binding categories. (PDF)

S1 Table Performance of predictive models trained and evaluated on separate sets. (PDF)

S2 Table Oligomerization of design-target complexes assayed by equilibrium analytical ultracentrifugation. (PDF)

S3 Table Calculated FRET efficiencies at 37°C between the designed peptide labeled with an acceptor fluorophore at the N- vs. C-terminus and a C-terminally labeled donor bZIP target. (PDF)

S4 Table Competition binding assay using an unlabeled designed peptide. (PDF)

S5 Table K_d_ values in nanomolar for JUN-d1 labeled at the C-terminus. (PDF)

S6 Table K_d_ values (nM) for ATF4-d1 labeled at the C-terminus, with notation as for S5 Table. (PDF)

S7 Table K_d_ values for XBP1-d1 (nM) labeled at the C-terminus, with notation as for S5 Table. (PDF)

S8 Table K_d_ values for ATF5-d1 (nM) labeled at the C-terminus, with notation as for S5 Table. (PDF)

S9 Table K_d_ values for JUN-d1 (nM) labeled at the N-terminus, with notation as for S5 Table. (PDF)

S10 Table K_d_ values for ATF4-d1 (nM) labeled at the N-terminus, with notation as for S5 Table. (PDF)

S11 Table K_d_ values for XBP1-d1 (nM) labeled at the N-terminus, with notation as for S5 Table. (PDF)

S12 Table K_d_ values for ATF5-d1 (nM) labeled at the N-terminus, with notation as for S5 Table. (PDF)

S1 Data All-vs.-all sequence identities of all native and designed proteins in this study. (XLS)

S2 Data Experimental dataset. (CSV)
