# Correction: Data-Driven Prediction and Design of bZIP Coiled-Coil Interactions

**DOI:** 10.1371/journal.pcbi.1004243

**Published:** 2015-04-15

**Authors:** The PLOS Computational Biology Staff

An error was introduced during the production process. A fixed-width font should have been used for the sequences in Table 3. The publisher apologizes for the error.

Please view the corrected table here:

**Table 3:** Binding of designed peptides to their bZIP targets

****Design****	****Target****	****K****_d_ ****at 37 °C (nM)****	****Designed sequence****
XBP1-d1 ^1^	XBP1	172 ± 65	EREAQLENRVAHLKEKNQELKAQNLHLKEALSEAQNRNQELKNDA
XBP1-d2	XBP1	≥ 5,000	AETDQLENRVKDLKKKNESLKEEKRQASNKYKALLTNNRSLKVKA
XBP1-d2* ^2^	XBP1	≥ 5,000 (269 at 23 °C)	AETDQLENRVKDLKKKNESLKEEKRQA**K**NK**L**KALLTNNRSLKVKA
JUN-d1 ^1^	JUN	5.8 ± 0.7	SIAATLEKEEANLEKMNKKLAAEIESLLKEKDKLESVLNYHE
JUN-d2	JUN	1	QRALQLQKEKERLEKMNKKLAAEIESLLEERERLESVLNYHE
ATF3-d1	ATF3	~1,000	NDLARLENKAEELKVQNRILVDERKYLQREISELHDELAAHE
ATF3-d2	ATF3	564	NLVAQLEKKNEALKAENAALEIERIQLQDKIEELKYELAAIE
ATF3-d3	ATF3	≥ 5,000 (117 at 4 °C)	KDAASLENKKEELKVQNRILVDERKYLQMMNSELKDELAAHE
ATF4-d1 ^1^	ATF4	9.3 ± 2.3	NQIKTLRTRLSKLRKDNLQLEKDIANLERKAKDLRAEKEQLEYEL
ATF5-d1 ^1^	ATF5	1680 ± 1050	KRIAYLRQRIAELRNENHVLESRIQRMEKEKDALQQDRDHLEYEL
ATF5-d1 ^3^	ATF4	4.9 ± 1.0	(same as above)

^1^ These four designs were chosen for further characterization.

^2^ A double mutant of XBP1-d2, in which serine at position 4e and tyrosine at position 5a were mutated to lysine and leucine, respectively.

^3^ Although ATF5 was used in the computational design, binding was tighter to ATF4, which shares 69.2% sequence identity with ATF5 in ***a***, ***d***, ***e*** and ***g*** positions.
