# Supplementary material for: Data-Driven Prediction and Design of bZIP Coiled-Coil Interactions
Source: PLoS Comput Biol. 2015 Feb 19;11(2):e1004046. doi: 10.1371/journal.pcbi.1004046 (PMC4335062; doi:10.1371/journal.pcbi.1004046)
Supplement: S1 Fig — (PDF) [file pcbi.1004046.s001.pdf]

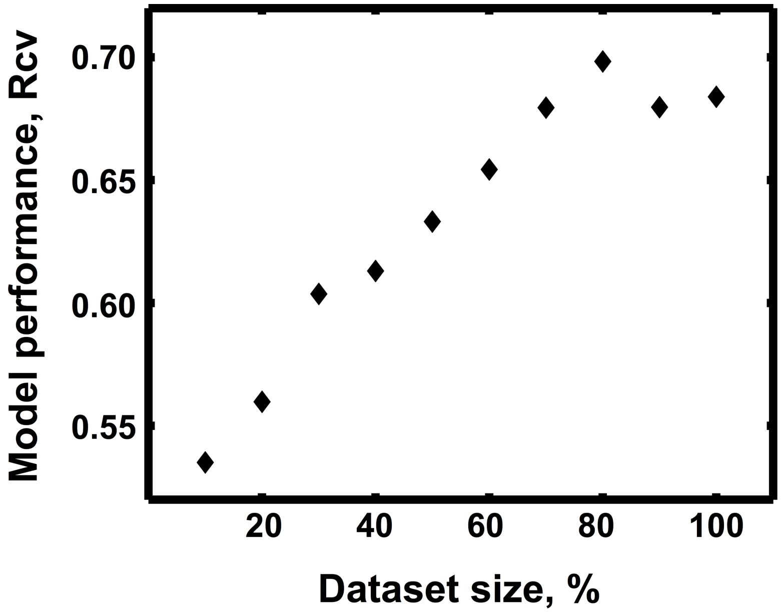

Figure S1. Model performance as a function of the amount of training data. An increasing fraction of the experimental data was used to train and evaluate a series of models, as described in the Methods. No further improvement in model performance was observed after ≥ 80% of the data were used to build the model.
