# Supplementary material for: Data-Driven Prediction and Design of bZIP Coiled-Coil Interactions
Source: PLoS Comput Biol. 2015 Feb 19;11(2):e1004046. doi: 10.1371/journal.pcbi.1004046 (PMC4335062; doi:10.1371/journal.pcbi.1004046)
Supplement: S2 Fig — (PDF) [file pcbi.1004046.s002.pdf]

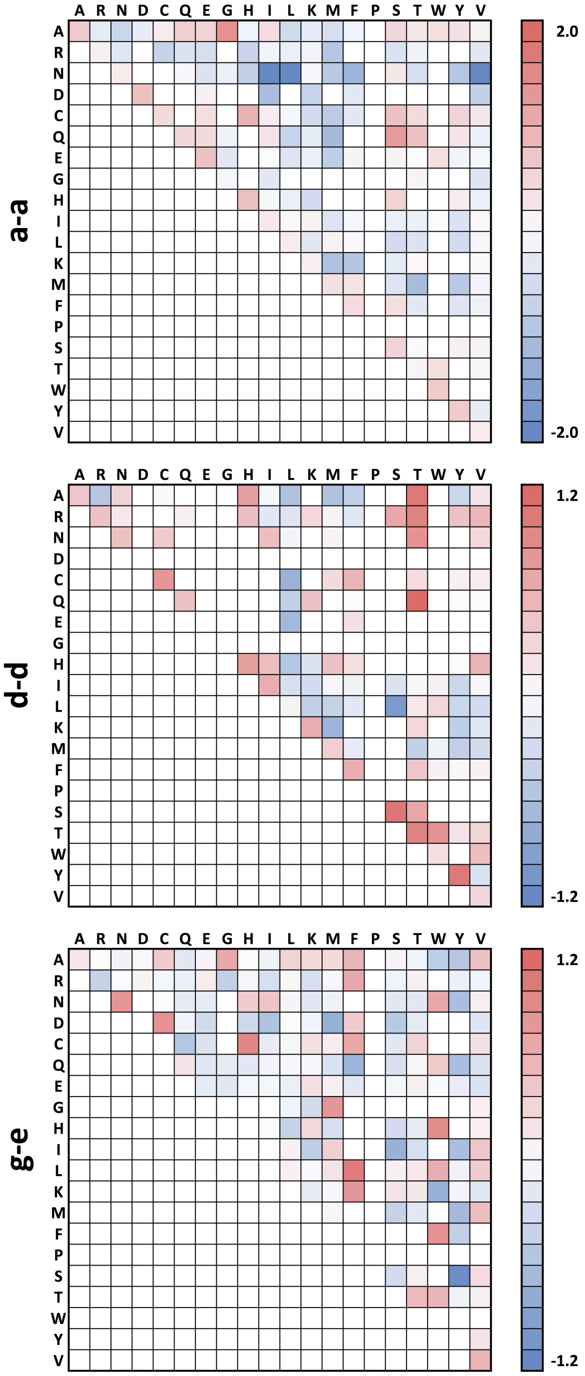

Figure S2. Differential heat maps of the 20 x 20 amino-acid interactions for a_i-a'_i, d_i-d'_i, g_i-e'_{i+1} pairs. Frequencies of all residue-residue interactions were calculated in binders ($1 < K_d < 5{,}000$ nM) and weak/non-binders ($K_d \geq 5{,}000$ nM) for $\mathbf{a_i}$-$\mathbf{a'_i}$, $\mathbf{d_i}$-$\mathbf{d'_i}$, $\mathbf{g_i}$-$\mathbf{e'_{i+1}}$ pairs. Then the log-ratios were calculated as $\log_{10}\left(f_{binder}^{aa} / f_{non-binder}^{aa}\right)$ and plotted as heat maps. Red colors correspond to interactions that are more frequent in binders; blue colors correspond to interactions that are more frequent in non-binders.
