# Supplementary material for: Data-Driven Prediction and Design of bZIP Coiled-Coil Interactions
Source: PLoS Comput Biol. 2015 Feb 19;11(2):e1004046. doi: 10.1371/journal.pcbi.1004046 (PMC4335062; doi:10.1371/journal.pcbi.1004046)
Supplement: S3 Fig — (PDF) [file pcbi.1004046.s003.pdf]

(a)

(b)

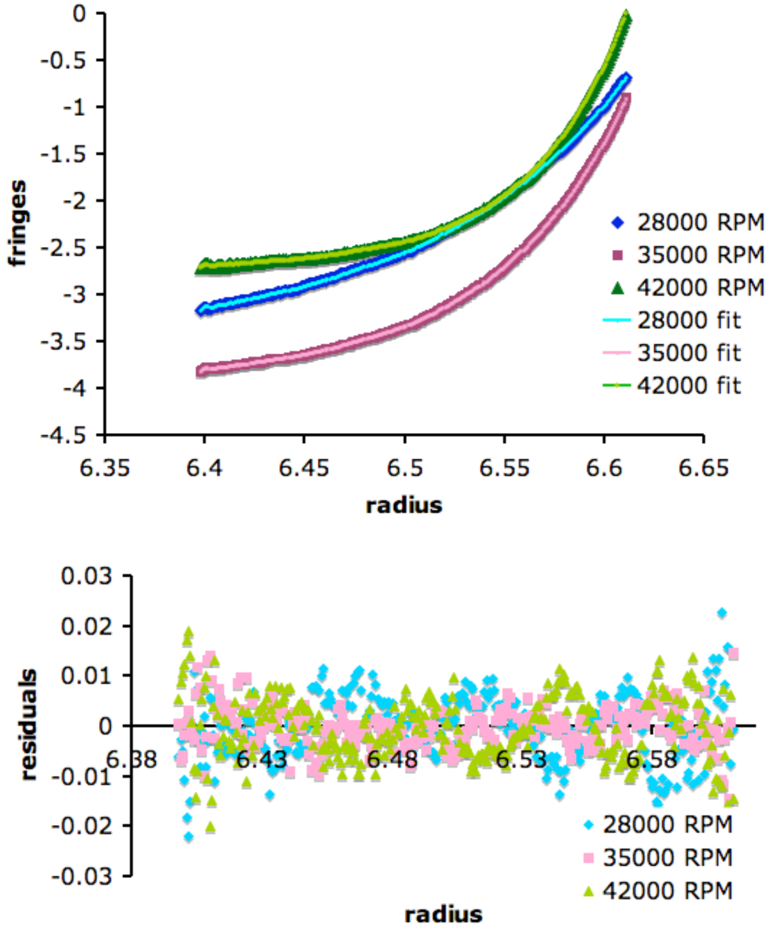

Figure S3. Analytical ultracentrifugation data for JUN-d1 mixed with JUN. (a) An example of the AUC data for an equimolar mixture of JUN-d1 and JUN (40 μM total protein concentration), at three different rotor speeds. The three speeds are plotted in blue, maroon, and dark green. The best-fit lines for each spin are shown in cyan, pink, and light green. (b) The residuals for the fit. The RMSD is 0.006 fringes
