# Supplementary material for: Data-Driven Prediction and Design of bZIP Coiled-Coil Interactions
Source: PLoS Comput Biol. 2015 Feb 19;11(2):e1004046. doi: 10.1371/journal.pcbi.1004046 (PMC4335062; doi:10.1371/journal.pcbi.1004046)
Supplement: S4 Fig — (PDF) [file pcbi.1004046.s004.pdf]

(a)

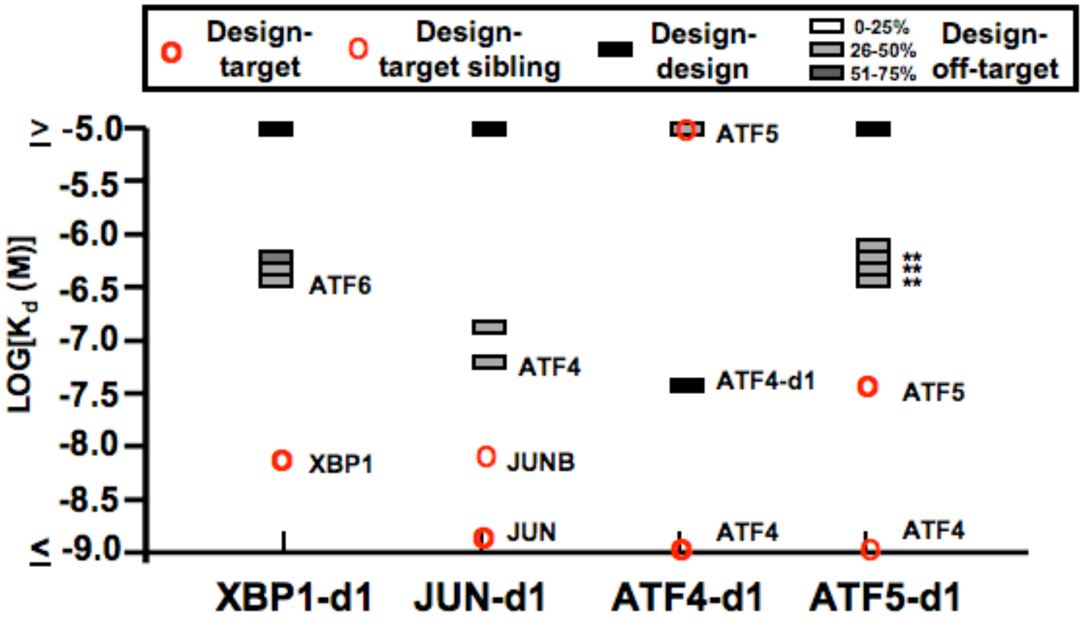

(b)

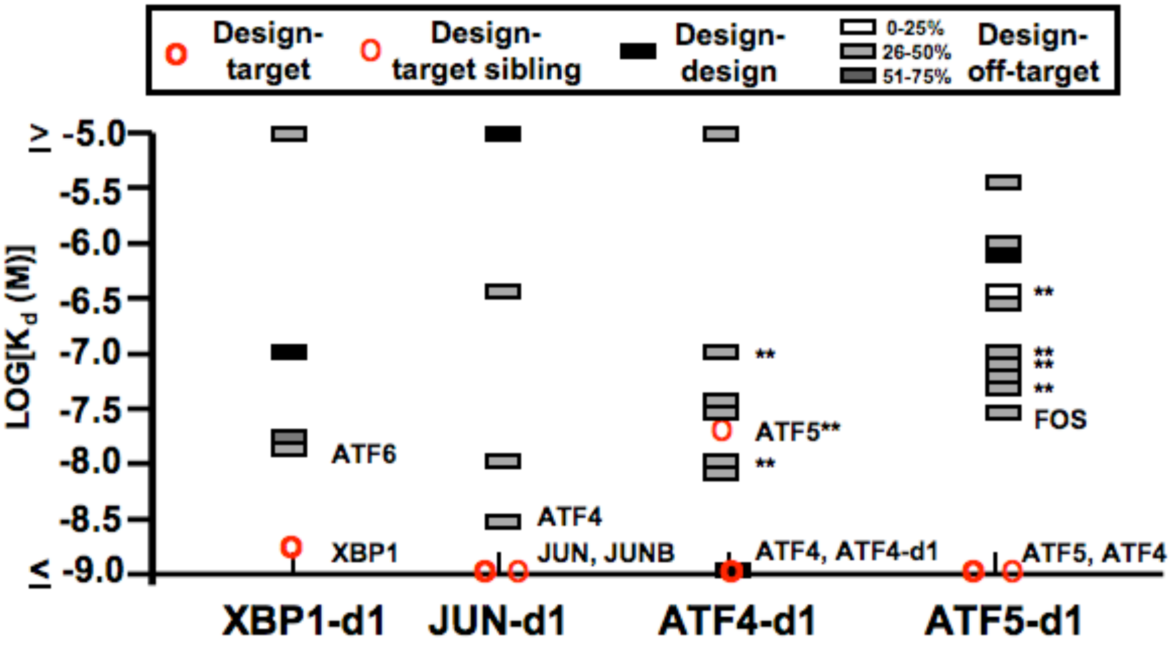

Figure S4. Specificity profiles of designed peptides XBP-d1, JUN-d1, ATF4-d1 and ATF5-d1. Dissociation constants were determined at (a) 23 °C and (b) 4 °C and plotted as in Figure 2. The sequence identity of each bZIP with the intended target is indicated (sequence identity is calculated using only core **a**, **d**, **e** and **g** residues). A double asterisk "**" indicates an interaction that was detected with the N-terminally labeled design. All K_d values are listed in Tables S5-S12.
