# Supplementary material for: Data-Driven Prediction and Design of bZIP Coiled-Coil Interactions
Source: PLoS Comput Biol. 2015 Feb 19;11(2):e1004046. doi: 10.1371/journal.pcbi.1004046 (PMC4335062; doi:10.1371/journal.pcbi.1004046)
Supplement: S5 Fig — (PDF) [file pcbi.1004046.s005.pdf]

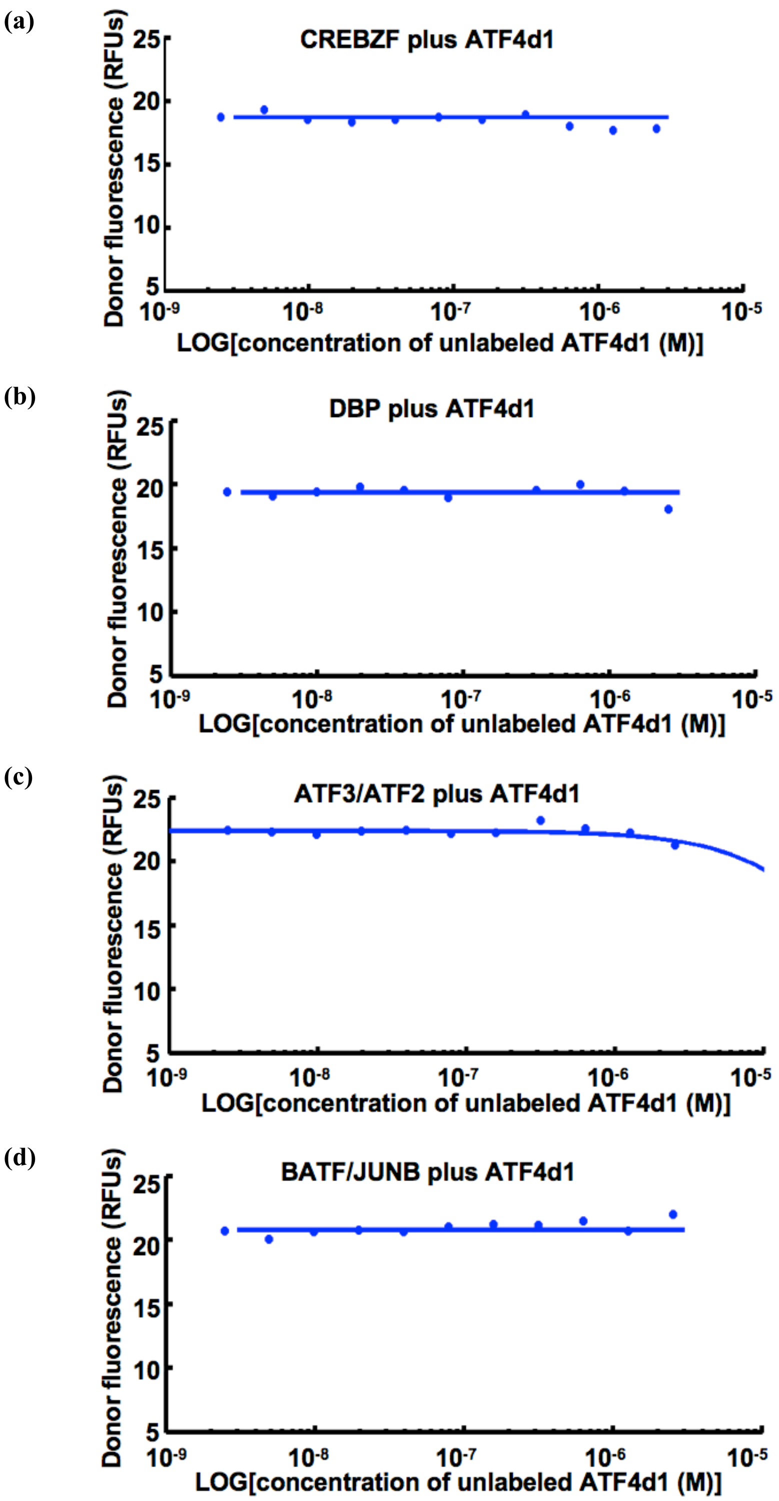

Figure S5. ATF4-d1 does not inhibit FRET complexes formed by CREBZF, DBP, ATF2 or BATF bZIP coiled coils. Native bZIP coiled coils were labeled with fluorescein or TAMRA. In the coiled-coil heterodimer FRET complexes, the donor-labeled FRET partner was the candidate partner of ATF4-d1. (a) CREBZF homodimer (10 nM fluorescein labeled plus 50 nM TAMRA labeled) plus unlabeled ATF4-d1. (b) DBP homodimer (10 nM fluorescein labeled plus 50 nM TAMRA labeled) plus unlabeled ATF4-d1. (c) 10 nM of ATF3 with 40 nM ATF2 plus unlabeled ATF4-d1. (d) 10 nM of BATF with 40 nM JUNB plus unlabeled ATF4-d1.
