# Supplementary material for: Data-Driven Prediction and Design of bZIP Coiled-Coil Interactions
Source: PLoS Comput Biol. 2015 Feb 19;11(2):e1004046. doi: 10.1371/journal.pcbi.1004046 (PMC4335062; doi:10.1371/journal.pcbi.1004046)
Supplement: S6 Fig — (PDF) [file pcbi.1004046.s006.pdf]

(a)

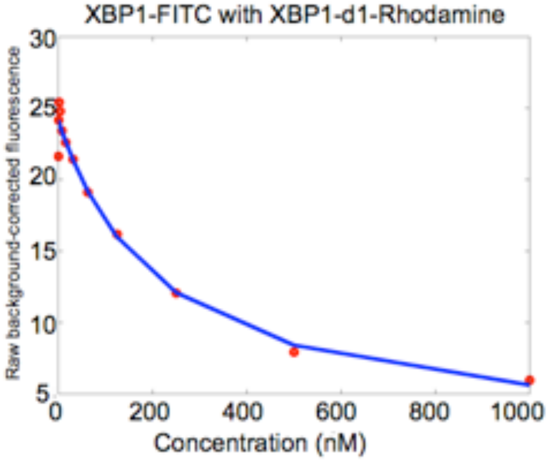
XBP1-FITC with XBP1-d1-Rhodamine
Raw background-corrected fluorescence
30
25
20
15
10
5
0
200
400
600
800
1000
Concentration (nM)

(b)

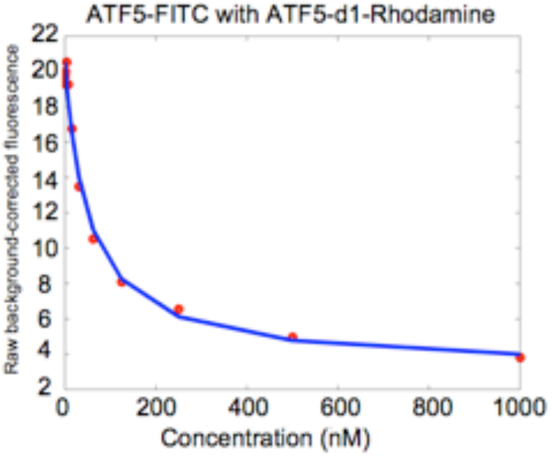
ATF5-FITC with ATF5-d1-Rhodamine
Raw background-corrected fluorescence
22
20
18
16
14
12
10
8
6
4
2
0
200
400
600
800
1000
Concentration (nM)

(c)

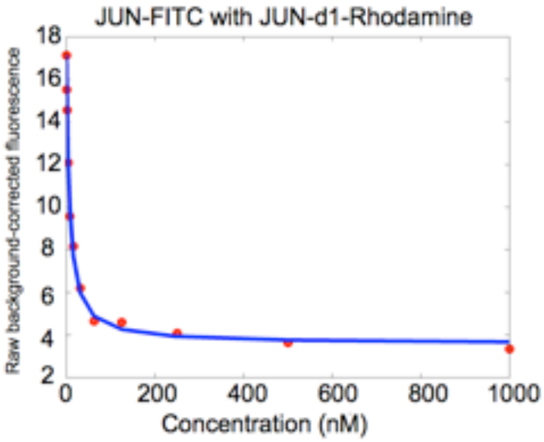
JUN-FITC with JUN-d1-Rhodamine
Raw background-corrected fluorescence
18
16
14
12
10
8
6
4
2
0
200
400
600
800
1000
Concentration (nM)

(d)

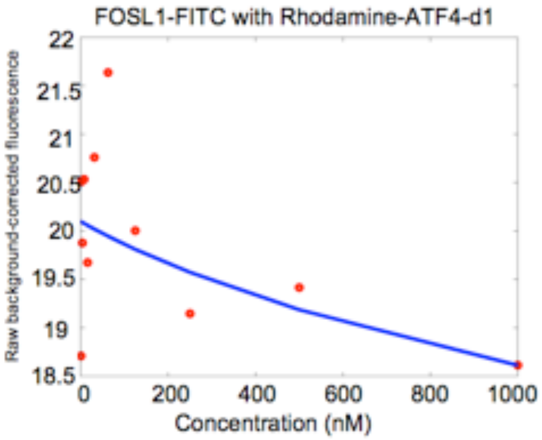
FOSL1-FITC with Rhodamine-ATF4-d1
Raw background-corrected fluorescence
22
21.5
21
20.5
20
19.5
19
18.5
0
200
400
600
800
1000
Concentration (nM)

(e)

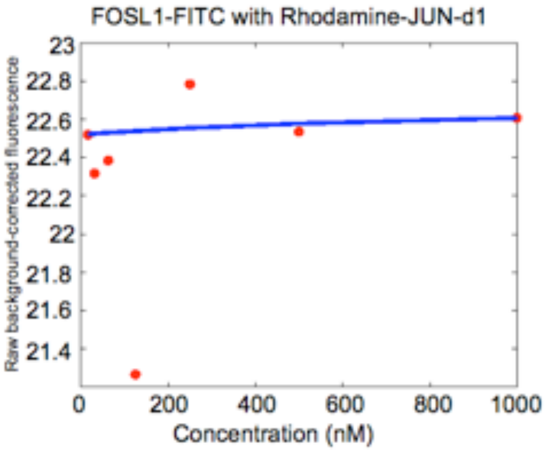
FOSL1-FITC with Rhodamine-JUN-d1
Raw background-corrected fluorescence
23
22.8
22.6
22.4
22.2
22
21.8
21.6
21.4
0
200
400
600
800
1000
Concentration (nM)

(f)

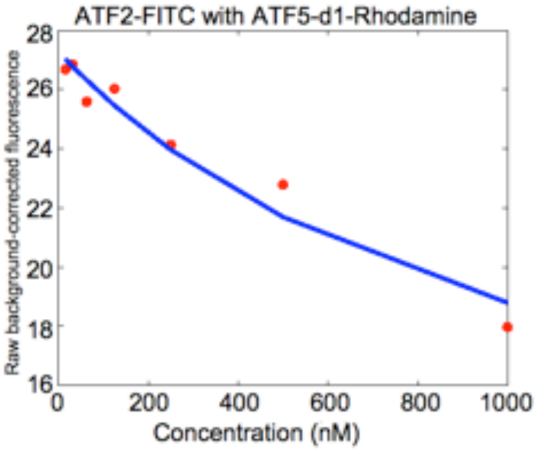
ATF2-FITC with ATF5-d1-Rhodamine
Raw background-corrected fluorescence
28
26
24
22
20
18
16
0
200
400
600
800
1000
Concentration (nM)

(g)

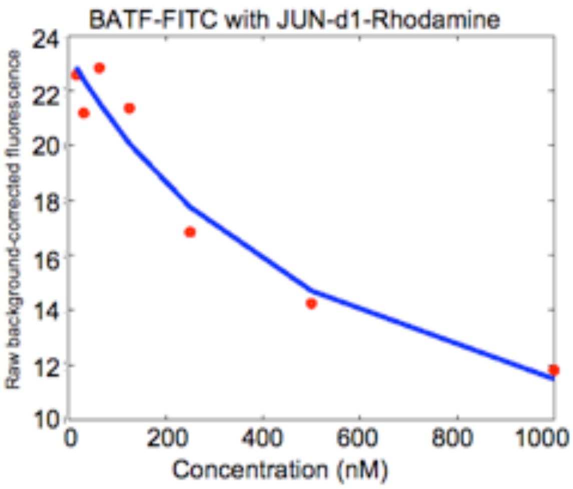

(h)

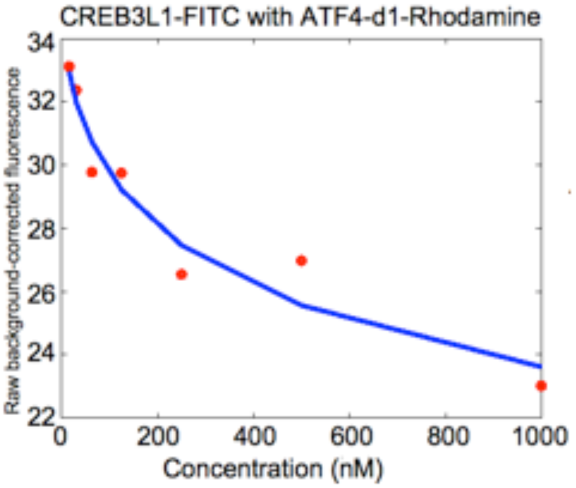

(i)

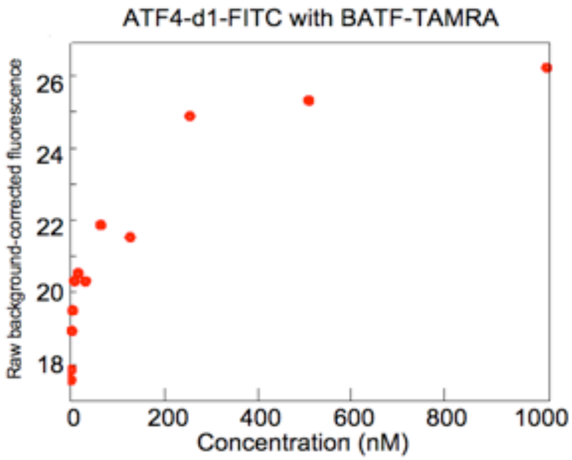

(j)

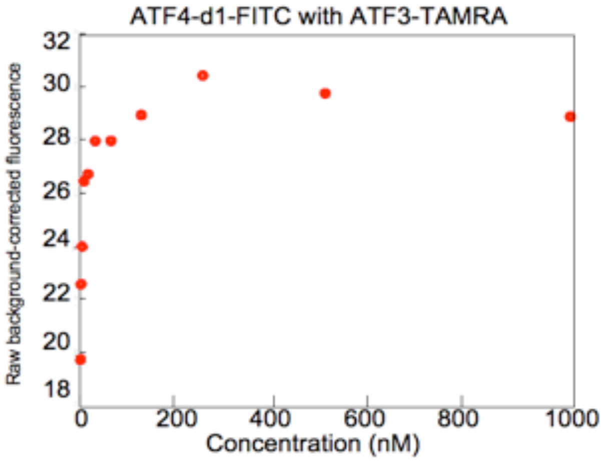

Figure S6. Representative plots of the different binding categories. The rhodamine-labeled designed peptide indicated was titrated into a fixed concentration of FITC-labeled target protein (a-h), or TAMRA-labeled target protein was titrated into a fixed concentration of FITC-labeled designed peptide (i-j). (a-c) Examples of fitted K_ds. (d) Lack of interaction classified as "NS." (e) An example of a re-fit curve showing no interaction and classified as "NI." (f) An example of a re-fit curve showing a weak interaction and classified as "AS-weak." (g) An example of a re-fit curve classified as "AS-moderate." (h) An example of a re-fit curve showing a stronger interaction and classified as "AS-strong." (i-j) Examples of curves that could not be fit using the fitting routine and were classified as "ND." For "NI" and all "AS" curves, the first 5 points that showed noisy, increasing donor fluorescence were removed and are not shown here. Fluorescence emission was monitored at 525 nm.
