# Supplementary material for: Data-Driven Prediction and Design of bZIP Coiled-Coil Interactions
Source: PLoS Comput Biol. 2015 Feb 19;11(2):e1004046. doi: 10.1371/journal.pcbi.1004046 (PMC4335062; doi:10.1371/journal.pcbi.1004046)
Supplement: S1 Table — (PDF) [file pcbi.1004046.s007.pdf]

Table S1. Performance of predictive models trained and evaluated on separate sets

Model	Number of features	Training set [a]		Test set	
		R	AUC	R	AUC
pairs & triplets	15,528 (773)	0.66 (0.66)	0.94 (0.94)	0.69 (0.68)	0.93 (0.92)
pairs (all)	1,893 (279)	0.63 (0.60)	0.92 (0.91)	0.63 (0.64)	0.89 (0.87)
pairs ($\mathbf{a_i a'_i}$, $\mathbf{d_i d'_i}$, $\mathbf{g_i e'_{i+1}}$)	507 (127)	0.55 (0.55)	0.89 (0.89)	0.62 (0.63)	0.88 (0.88)

To obtain the training and test sets, the original dataset was divided into 10 non-overlapping partitions as described in the Methods. The first 7 partitions (70% of data) were merged into the training set, and the remaining 3 partitions (30% of data) were merged into the test set. Numbers in parentheses refer to performance with a reduced feature set, as in Table 2.

[a] Performance on the training set was assessed using the same 10-fold nested cross-validation protocol as was used in Table 1 for the entire data set, but with fewer interactions (70% of the total interactions). The best resulting model was used to make predictions on the test set.
