# Supplementary material for: Data-Driven Prediction and Design of bZIP Coiled-Coil Interactions
Source: PLoS Comput Biol. 2015 Feb 19;11(2):e1004046. doi: 10.1371/journal.pcbi.1004046 (PMC4335062; doi:10.1371/journal.pcbi.1004046)
Supplement: S2 Table — (PDF) [file pcbi.1004046.s008.pdf]

Table S2. Oligomerization of design-target complexes assayed by equilibrium analytical ultracentrifugation

Complex	Fitted mass	Expected mass	Ratio (fitted/expected for a heterodimer)	RMSD [a]
XBP1-d1 / XBP1	15,503	15,137	1.02	0.013
JUN-d1 / JUN	15,498	14,093	1.1	0.006
ATF4-d1 / ATF4	17,509	15,128	1.16	0.013
ATF5-d1 / ATF5	16,226	15,314	1.06	0.008

Complexes between XBP1-d1, JUN-d1, and ATF5-d1 and the intended target were formed at 40 μM total peptide. The complex between ATF4-d1 and ATF4 was formed at 80 μM total peptide. See Methods for details of the experiment.

[a] RMSD reports the difference between the data and the fit, in fringes.
