# Supplementary material for: Data-Driven Prediction and Design of bZIP Coiled-Coil Interactions
Source: PLoS Comput Biol. 2015 Feb 19;11(2):e1004046. doi: 10.1371/journal.pcbi.1004046 (PMC4335062; doi:10.1371/journal.pcbi.1004046)
Supplement: S3 Table — (PDF) [file pcbi.1004046.s009.pdf]

Table S3. Calculated FRET efficiencies at 37 ºC between the designed peptide labeled with an acceptor fluorophore at the N- vs. C-terminus and a C-terminal labeled donor bZIP target

Complex	FRET efficiency (label on design C-terminus)	FRET efficiency (label on design N-terminus)
XBP1-d1 / XBP1	0.79	0.35
JUN-d1 / JUN	0.76	0.52
ATF4-d1 / ATF4	0.87	0.35
ATF5-d1 / ATF4	0.54	0.38
ATF5-d1/ATF5	0.42	0.2

See Methods for details.
