# Supplementary material for: Data-Driven Prediction and Design of bZIP Coiled-Coil Interactions
Source: PLoS Comput Biol. 2015 Feb 19;11(2):e1004046. doi: 10.1371/journal.pcbi.1004046 (PMC4335062; doi:10.1371/journal.pcbi.1004046)
Supplement: S4 Table — (PDF) [file pcbi.1004046.s010.pdf]

Table S4. Competition binding assay using an unlabeled designed peptide

Complex	K_I (nM)
ATF4-d1 / ATF4	48
ATF5-d1 / ATF4	6

The unlabeled design was mixed with FITC-labeled design and TAMRA-labeled ATF4. See Methods for details.
