# Supplementary material for: Data-Driven Prediction and Design of bZIP Coiled-Coil Interactions
Source: PLoS Comput Biol. 2015 Feb 19;11(2):e1004046. doi: 10.1371/journal.pcbi.1004046 (PMC4335062; doi:10.1371/journal.pcbi.1004046)
Supplement: S5 Table — (PDF) [file pcbi.1004046.s011.pdf]

Table S5. K_d values in nanomolar for JUN-d1 labeled at the C-terminus

	**37 °C**	**23 °C**	**4 °C**
**FOS**	(NS, ≥5000)[1] (NS)[2]	(NS, ~1000)[1] (NS)[2]	(≥5000, ~1000)[1] (NS)[2]
**FOSL1**	NI	NI	NS
**JUN**	**5.8** (6.6, 5.3, 5.5)[1] (16.1)[2]	**1.1** (1.2, 1.2, 1.0)[1] (4.41)[2]	**1.0** (1.0, 1.0, 1.0)[1] (2.1)[2]
**JUNB**	**51**	**8.4**	1
**MAF**	NI	NI	NI
**MAFB**	NS	NS	**326**
**MAFF**	(NS, ≥5000)[1]	(NS, ~1000)[1]	(NS, ~1000)[1]
**MAFG**	(NI ,≥5000)[1]	(NS, ~1000)[1]	(NS, ~1000)[1]
**ATF2**	**716** (680.6, 760.1, ≥5000, 708)[1]	**108** (109.6, 104.2, 106.8, 112)[1]	**12** (12.0, 12.1, 14.0, 9.7)[1]
**ATF3**	(NS, ≥5000)[1] (NS)[2]	(NI, ~1000)[1] (NS)[2]	(≥5000, ~1000)[1] (NS)[2]
**ATF4**	**543** (556.9, 529.4, 542.8)[1] (441.2)[2]	**50** (52.1, 54.3, 44.3)[1] (40.1)[2]	**3.5** (3.7, 3.3, 3.6)[1] (6.91)[2]
**ATF5**	NS (NS)[2]	NS (NS)[2]	≥5000 (NS)[2]
**ATF6**	NS (NS)[2]	NS (NS)[2]	NI (NI)[2]
**ATF6B**	(NS, ≥5000)[1] (NS)[2]	(NS, ~1000)[1] (NS)[2]	(NI, ~1000)[1] (AS-moderate*)[2]
**CREBZF**	NS (NI)[2]	NS (NI)[2]	NI (NI)[2]
**XBP1**	(NS, ≥5000)[1]	(NS, ~1000)[1]	(NS, ~1000)[1]
**NFE2**	(NS, ≥5000)[1] (NS)[2]	(NS, ~1000)[1] (NS)[2]	(NS, ~1000)[1] (NS)[2]
**NFE2L1**	AS-weak	AS-weak	AS-weak
**NFE2L2**	AS-weak (NI)[2]	NS (NI)[2]	NS (NI)[2]
**NFE2L3**	(NI , ≥5000)[1]	(NS, ~1000)[1]	(NS, ~1000)[1]
**CREB1**	(NS, ≥5000)[1] (NI)[2]	(NS, ~1000)[1] (NI)[2]	(NS, ~1000)[1] (NI)[2]
**CREB3**	NI	NI	NI
**CREB3L1**	NS	NS	NS
**CREB3L3**	NS (NS)[2]	NS (NS)[2]	NS (NS)[2]
**BACH1**	NS (NS)[2]	NS (NS)[2]	NS (NS)[2]
**BACH2**	NS (NS)[2]	NS (NS)[2]	AS-weak (NS)[2]
**BATF**	NS (NS)[2]	NS (NS)[2]	AS-moderate (NS)[2]
**BATF2**	NS (NS)[2]	NS (NS)[2]	AS-weak (NS)[2]
**BATF3**	NS	AS-weak	**362**
**HLF**	NS (NI)[2]	NS (NI)[2]	NS (NI)[2]
**DBP**	NS (NS)[2]	AS-weak (NI)[2]	≥5000 (NI)[2]
**NFIL3**	NS (NS)[2]	NS (NS)[2]	NS (NS)[2]
**homodimer**	NS	NS	NS

Notes for Tables S5 – S12:

The average K_d is listed in bold, with individual measurements in parentheses. Averages excluding an outlier are shown in italics. See Methods for details on the fitting procedure.

[1] K_d values for repeated measurements in which the designed peptide was labeled with the FRET acceptor (rhodamine) and titrated into the donor-labeled target.

[2] K_d value for measurements in which the designed peptide was labeled with the FRET donor (fluorescein) and acceptor-labeled target was titrated to increasing concentrations.

"*" denotes an R^2 value < 0.8.

"NS" denotes little or no signal change; interpreted as no binding.

For some curves, the signal increased over the first few titration points. For these curves, the first 5 points were removed and the curve was re-evaluated, leading to one of four categories (see Methods):

"NI" denotes noisy data after removal of the first 5 points, interpreted as no interaction.

"AS-weak" denotes an interaction with $K_d \geq 5000$ nM after removing the first 5 points and re-fitting

"AS-moderate" denotes an interaction with $200 < K_d < 1000$ nM after removing the first 5 points and re-fitting.

"AS-strong" denotes an interaction with $K_d < 200$ nM after removing the first 5 points and re-fitting.

"ND" denotes an interaction with K_d not determined due to a continuous increase in donor fluorescence signal with increasing acceptor; this likely corresponds to some interaction in a mode that does not fit our binding model.
