# Supplementary material for: Data-Driven Prediction and Design of bZIP Coiled-Coil Interactions
Source: PLoS Comput Biol. 2015 Feb 19;11(2):e1004046. doi: 10.1371/journal.pcbi.1004046 (PMC4335062; doi:10.1371/journal.pcbi.1004046)
Supplement: S6 Table — (PDF) [file pcbi.1004046.s012.pdf]

Table S6. K_d values (nM) for ATF4-d1 labeled at the C-terminus, with notation as for Table S5

	**37 °C**	**23 °C**	**4 °C**
**FOS**	(NS, ≥5000)[1] (NI)[2]	(AS-weak, ~1000)[1] (NS)[2]	~1000 (~1000, ~1000)[1] (1000)[2]
**FOSL1**	NS (NI)[2]	NS (NI)[2]	NS (≥5000)[2]
**JUN**	**623** (NS)[2]	AS-strong (NS)[2]	AS-strong (NS)[2]
**JUNB**	NS (NS)[2]	NS (NS)[2]	NI (NI)[2]
**MAF**	**460**	≥5000	≥5000
**MAFB**	**402**	≥5000	≥5000
**MAFF**	(AS-strong, AS-strong, 326.6)[1]	(AS-weak, AS-strong, 622.9)[1]	≥5000 (≥5000, ≥5000, ≥5000)[1]
**MAFG**	(AS-moderate ,≥5000)[1]	(AS-weak ,~1000)[1]	(AS-weak ,1000)[1]
**ATF2**	(AS-weak, ≥5000)[1] (AS-weak)[2]	(NS, ~1000)[1] (NS)[2]	(AS-weak ,1000) (AS-weak)[2]
**ATF3**	(NS, ≥5000)[1] (NS)[2]	(NS, ~1000)[1] (NS)[2]	(NI, 1000) (ND)[2]
**ATF4**	**9.3** (11.3, 9.9, 6.8)[1] (10.0)[2]	**1.0** (1.0, 1.0, 1.0)[1] (3.7)[2]	**1.0** (1.0, 1.0, 1.0)[1] (2.3)[2]
**ATF5**	AS-weak (AS-weak, AS-weak AS-weak)[1] (NS)[2]	AS-weak (AS-weak, AS-weak, AS-weak)[1] (~1000)[2]	≥5000 (≥5000, ≥5000, 473.91) (35.5)[2]
**ATF6**	NS (NS)[2]	NS (NS)[2]	NS (NS)[2]
**ATF6B**	(AS-weak, ≥5000)[1] (NS)[2]	(AS-weak, ~1000)[1] (NS)[2]	(AS-weak, ~1000)[1] (NI)[2]
**CREBZF**	NS (NS)[2]	AS-weak (NS)[2]	AS-weak (ND)[2]
**XBP1**	(NS, ≥5000)[1]	(NI, 1000)	(NS, ~1000)[1]
**NFE2**	(AS-weak , AS-weak, 2917.6)[1] (NS)[2]	(AS-moderate, ≥5000, ≥5000)[1] (1000)[2]	**31** (51.9, 10.9, 3.4)[1] (42.4)[2]
**NFE2L1**	NS	AS-weak	≥5000
**NFE2L2**	(NS, NS, AS-weak)[1] (NS)[2]	AS-weak (AS-weak, AS-weak, AS-weak)[1] (NS)[2]	**10** (11.6, 12.0, 6.8)[1] (AS-weak)[2]
**NFE2L3**	(AS-weak ,≥5000)[1]	(AS-weak ,~1000)	(AS-weak ,~1000)[1]
**CREB1**	(AS-weak, ≥5000)[1] (NS)[2]	(NS, ~1000) (NS)[2]	(NS, ~1000)[1] (AS-weak*)[2]
**CREB3**	AS-weak	NI	NS
**CREB3L1**	NS	AS-strong	≥5000
**CREB3L3**	NS (NS)[2]	NS (NS)[2]	AS-weak (NS)[2]
**BACH1**	NS (NS)[2]	NS (NS)[2]	≥5000 (NS)[2]
**BACH2**	AS-weak (NS)[2]	NS (AS-weak)[2]	AS-weak (NS)[2]
**BATF**	NS (NS)[2]	NS (NS)[2]	NS (ND)[2]
**BATF2**	NS (NS)[2]	NS (NS)[2]	NS (NS)[2]
**BATF3**	NS	NS	NS
**HLF**	AS-weak (NS)[2]	NS (NS)[2]	AS-weak (NS)[2]
**DBP**	NS (NS)[2]	NS (NS)[2]	AS-weak (ND)[2]
**NFIL3**	AS-weak (AS-weak, AS-weak, AS-weak*)[1] (NS)[2]	(AS-weak, AS-weak, ≥5000)[1] (NS)[2]	**22.8** (23.3, 22.6, 22.6)[1] (42.4)[2]
**homodimer**	**1816** (1110.4, 2522)[1]	**36.3** (57.1 15.5)[1]	**1.0** (1.0, 1.0)[1]
