# Supplementary material for: Data-Driven Prediction and Design of bZIP Coiled-Coil Interactions
Source: PLoS Comput Biol. 2015 Feb 19;11(2):e1004046. doi: 10.1371/journal.pcbi.1004046 (PMC4335062; doi:10.1371/journal.pcbi.1004046)
Supplement: S7 Table — (PDF) [file pcbi.1004046.s013.pdf]

**Table S7.** $K_d$ values for XBP1-d1 (nM) labeled at the C-terminus, with notation as for Table S5.

| | **37 °C** | **23 °C** | **4 °C** |
|---|---|---|---|
| **FOS** | NS (NS)[2] | NS (NS)[2] | NS (NS)[2] |
| **FOSL1** | NS | NS | NI |
| **JUN** | NS | NS | NS |
| **JUNB** | NS | NS | NS |
| **MAF** | NS (NS)[2] | NS (NS)[2] | NS (NI)[2] |
| **MAFB** | AS-weak*(NS)[2] | AS-weak* (NS)[2] | AS-weak* (NS)[2] |
| **MAFF** | NS (NS)[2] | NS (NS)[2] | NS (NS)[2] |
| **MAFG** | NS (NS)[2] | NS (NS)[2] | NI (NS)[2] |
| **ATF2** | NS (NS)[2] | NS (NS)[2] | NS (NS)[2] |
| **ATF3** | NS (NS)[2] | NS (NS)[2] | NI (NS)[2] |
| **ATF4** | NS | NS | NS |
| **ATF5** | NS | NS | NS |
| **ATF6** | AS-weak (AS-weak, AS-weak, AS-weak*)[1] (NS)[2] | **397** (331.0, 305.9, 553.74)[1] (NS)[2] | **12.6** (15.7, 15.6, 6.6)[1] (AS-weak)[2] |
| **ATF6B** | NS (NS, NS)[1] (NS)[2] | **458** (583.6, 332.9)[1] (NS)[2] | (AS-moderate, NI)[1] (NS)[2] |
| **CREBZF** | NS (NS NS NS)[1] (NI)[2] | **492** (335.3, 383.5, 757.9)[1] (NI)[2] | **20.2** (18.6, 23.8, 18.2)[1] (NS)[2] |
| **XBP1** | **172** (207.9, 226.8, 170.9, 80.2)[1] | **7.0** (9.4, 10.5, 5.8, 2.4)[1] | **1.8** (2.3, 2.0, 1.7, 1.0)[1] |
| **NFE2** | NS (NS)[2] | NS (NS)[2] | NS (NI)[2] |
| **NFE2L1** | NS (NS)[2] | NS (NS)[2] | NS (NS)[2] |
| **NFE2L2** | NS (NS)[2] | NS (NI)[2] | NI (NI)[2] |
| **NFE2L3** | AS-weak* | NS | NS |
| **CREB1** | NS | NS | $\geq$5000 |
| **CREB3** | NS | NI | NI |
| **CREB3L1** | NS (NS)[2] | NS (NS)[2] | NI (NS)[2] |
| **CREB3L3** | NS | NS | NI |
| **BACH1** | NS (NS)[2] | NS (NS)[2] | NS (NS)[2] |
| **BACH2** | NS (NS)[2] | NS (NS)[2] | NS (NS)[2] |
| **BATF** | NS (NS)[2] | NS (NS)[2] | $\geq$5000 (AS-weak)[2] |
| **BATF2** | NS | NS | AS-strong |
| **BATF3** | NS | NS | $\geq$5000 |
| **HLF** | NS (NS)[2] | NI (NI)[2] | NI (NI)[2] |
| **DBP** | NS (NS)[2] | NI (NS)[2] | NI (NS)[2] |
| **NFIL3** | NS (NS)[2] | NS (NS)[2] | NS (NS)[2] |
| **homodimer** | NS | NS | **110** |
