# Supplementary material for: Data-Driven Prediction and Design of bZIP Coiled-Coil Interactions
Source: PLoS Comput Biol. 2015 Feb 19;11(2):e1004046. doi: 10.1371/journal.pcbi.1004046 (PMC4335062; doi:10.1371/journal.pcbi.1004046)
Supplement: S8 Table — (PDF) [file pcbi.1004046.s014.pdf]

Table S8. K_d values for ATF5-d1 (nM) labeled at the C-terminus, with notation as for Table S5.

	**37 °C**	**23 °C**	**4 °C**
**FOS**	(AS-weak, AS-weak, ≥5000)[1]	**391** (546.3, 393.3, 234.0)[1]	**37.9** (49.3, 41.4, 22.9)[1]
**FOSL1**	NS	**695**	**84.1**
**JUN**	≥5000	≥5000	**119**
**JUNB**	NS	NS	**3620**
**MAF**	NS	AS-weak	**790**
**MAFB**	NS	AS-weak	**725**
**MAFF**	AS-weak	≥5000	**266**
**MAFG**	NS	NS	AS-weak
**ATF2**	AS-weak	AS-weak	≥5000
**ATF3**	NS	AS-weak	≥5000
**ATF4**	**4.9** (4.8, 4.0, 6.0)[1]	**1.0** (1.0, 1.0, 1.0)[1]	**1.0** (1.0, 1.0, 1.0)[1]
**ATF5**	**1680** (2801.5, 1513.2, 729.3, 128.9)[1]	**44.1** (51.6, 49.2, 31.6, 9.3)[1]	**1.1** (1.1, 1.2, 1.0, 1.0)[1]
**ATF6**	NS	NS	AS-weak
**ATF6B**	(AS-weak, AS-weak, 260.34)[1]	(AS-weak*, AS-weak*, 108.0)[1]	**433** (447.5, 437.6, 412.3)[1]
**CREBZF**	NS	≥5000	**409**
**XBP1**	NS	NS	AS-weak
**NFE2**	≥5000	≥5000	**162**
**NFE2L1**	≥5000	≥5000	≥5000
**NFE2L2**	AS-weak	NS	AS-weak
**NFE2L3**	**911**	NS	≥5000
**CREB1**	~1000	**589**	**133**
**CREB3**	AS-moderate	≥5000	**147**
**CREB3L1**	~1000	**529**	**158**
**CREB3L3**	**657**	**414**	**230**
**BACH1**	AS-weak	NS	NS
**BACH2**	≥5000	AS-weak	≥5000
**BATF**	AS-weak	AS-weak	AS-weak
**BATF2**	NS	AS-weak	**591**
**BATF3**	≥5000	**791**	**192**
**HLF**	≥5000	AS-weak	AS-weak
**DBP**	NS	NS	NS
**NFIL3**	NS	NS	NS
**homodimer**	NS	~1000	~1000
