# Supplementary material for: Data-Driven Prediction and Design of bZIP Coiled-Coil Interactions
Source: PLoS Comput Biol. 2015 Feb 19;11(2):e1004046. doi: 10.1371/journal.pcbi.1004046 (PMC4335062; doi:10.1371/journal.pcbi.1004046)
Supplement: S9 Table — (PDF) [file pcbi.1004046.s015.pdf]

Table S9. K_d values for JUN-d1 (nM) labeled at the N-terminus, with notation as for Table S5.

	**37 °C**	**23 °C**	**4 °C**
**FOS**	NS	NI	NI
**FOSL1**	NS	NI	NI
**JUN**	**41.0** (48.1, 50.6, 35.8, 29.5)[1]	**5.2** (4.7, 7.3, 6.0, 2.8)[1]	**1.6** (1.5, 1.3, 2.3, 1.1)[1]
**JUNB**	**850**	**53**	**2.7**
**MAF**	NS	NS	NS
**MAFB**	NS	NS	NS
**MAFF**	NS	NS	NI
**MAFG**	NS	NS	NI
**ATF2**	NS	NS	AS-moderate
**ATF3**	NS	NS	NS
**ATF4**	NS	AS-weak	**592**
**ATF5**	NS	NS	NS
**ATF6**	NS	NS	NS
**ATF6B**	NS	NS	NS
**CREBZF**	NS	NS	NI
**XBP1**	NS	NS	NI
**NFE2**	NS	NI	NI
**NFE2L1**	NS	NS	NI
**NFE2L2**	NS	NS	NS
**NFE2L3**	NS	NS	NI
**CREB1**	NS	NS	NS
**CREB3**	NS	NS	NI
**CREB3L1**	NS	NS	NS
**CREB3L3**	NS	NS	NI
**BACH1**	NS	NI	NI
**BACH2**	NS	NS	NI
**BATF**	NS	NS	NS
**BATF2**	NS	NS	NI
**BATF3**	NS	NS	NS
**HLF**	NS	NS	NI
**DBP**	NS	NS	NI
**NFIL3**	NS	NS	NI
