# Supplementary material for: Data-Driven Prediction and Design of bZIP Coiled-Coil Interactions
Source: PLoS Comput Biol. 2015 Feb 19;11(2):e1004046. doi: 10.1371/journal.pcbi.1004046 (PMC4335062; doi:10.1371/journal.pcbi.1004046)
Supplement: S10 Table — (PDF) [file pcbi.1004046.s016.pdf]

Table S10. K_d values for ATF4-d1 (nM) labeled at the N-terminus, with notation as for Table S5.

	**37 °C**	**23 °C**	**4 °C**
**FOS**	NS	NS	132.8
**FOSL1**	NS	NS	AS-weak
**JUN**	AS-weak	AS-weak	NS
**JUNB**	NS	NS	NS
**MAF**	NS	NS	NS
**MAFB**	NS	NS	NS
**MAFF**	NS	NS	AS-strong
**MAFG**	NS	NS	NS
**ATF2**	NS	NS	NS
**ATF3**	NS	NS	NS
**ATF4**	**741** (~1000, ~1000, 635.4, 327.9)[1]	***235*** (490.3, 112.5, 102.7, 22.4)[1]	(AS-weak, AS-weak, ~1000, 71.5)[1]
**ATF5**	NS (NS, NS, NS)[1]	(NS, ≥5000, ≥5000)[1]	**15.5** (18.9, 18.7, 8.8)[1]
**ATF6**	NS	NS	NI
**ATF6B**	NS	NS	NI
**CREBZF**	NS	AS-weak	AS-strong
**XBP1**	NS	NS	NS
**NFE2**	NS	NS	AS-weak
**NFE2L1**	NS (NS, NS)[1]	NS (NS, NS)[1]	(NS, NI)[1]
**NFE2L2**	NS	NS	**47.9**
**NFE2L3**	NS	NI	NI
**CREB1**	NS	AS-weak	AS-weak
**CREB3**	NS	NS	NS
**CREB3L1**	NS	NS	NS
**CREB3L3**	NS	NS	NS
**BACH1**	NS (NS, NS)[1]	(NS, AS-weak)[1]	**12.3** (16.9, 7.6)[1]
**BACH2**	NS	AS-weak	NS
**BATF**	NS	NS	AS-weak
**BATF2**	NS	NS	NS
**BATF3**	NS	NS	NS
**HLF**	NS	NS	NS
**DBP**	NS	NS	NS
**NFIL3**	NS	NS	NS
