# Supplementary material for: Data-Driven Prediction and Design of bZIP Coiled-Coil Interactions
Source: PLoS Comput Biol. 2015 Feb 19;11(2):e1004046. doi: 10.1371/journal.pcbi.1004046 (PMC4335062; doi:10.1371/journal.pcbi.1004046)
Supplement: S11 Table — (PDF) [file pcbi.1004046.s017.pdf]

Table S11. K_d values for XBP1-d1 (nM) labeled at the N-terminus, with notation as for Table S5.

	**37 °C**	**23 °C**	**4 °C**
FOS	NS	NS	NI
**FOSL1**	NS	NS	NI
**JUN**	NS	NS	NS
**JUNB**	NS	NS	NS
**MAF**	NS	NS	NS
**MAFB**	NS	NS	NI
**MAFF**	NS	NS	NI
**MAFG**	NS	NS	NI
**ATF2**	NS	NS	NS
**ATF3**	NS	NS	NI
**ATF4**	NS	NS	NS
**ATF5**	NS	NS	NS
**ATF6**	NS	NS	ND
**ATF6B**	NS	NS	ND
**CREBZF**	NS	NS	NI
**XBP1**	**857** (~1000, ~1000, 570.2)[1]	(NS, NS , NS, ND)[1]	ND (ND, ND, ND , ND)[1]
**NFE2**	NI	NI	NI
**NFE2L1**	NS	NS	NI
**NFE2L2**	NS	NS	NS
**NFE2L3**	NS	NS	NI
**CREB1**	NS	NI	NI
**CREB3**	NS	NI	NI
**CREB3L1**	NS	NS	NI
**CREB3L3**	NS	NS	NI
**BACH1**	NS	NS	≥5000
**BACH2**	NS	NS	NS
**BATF**	NS	NS	NS
**BATF2**	NS	NS	NS
**BATF3**	NS	NS	NS
**HLF**	NS	NS	NI
**DBP**	NI	NI	NI
**NFIL3**	NS	NS	NI
