# Supplementary material for: Data-Driven Prediction and Design of bZIP Coiled-Coil Interactions
Source: PLoS Comput Biol. 2015 Feb 19;11(2):e1004046. doi: 10.1371/journal.pcbi.1004046 (PMC4335062; doi:10.1371/journal.pcbi.1004046)
Supplement: S12 Table — (PDF) [file pcbi.1004046.s018.pdf]

Table S12. K_d values for ATF5-d1 (nM) labeled at the N-terminus, with notation as for Table S5.

	**37 °C**	**23 °C**	**4 °C**
**FOS**	≥5000	**753**	**89.6**
**FOSL1**	NI	≥5000	**271**
**JUN**	AS-weak	AS-weak	≥5000
**JUNB**	NS	NS	NS
**MAF**	NS	≥5000	≥5000
**MAFB**	≥5000	AS-weak	≥5000
**MAFF**	(AS-weak, 115.6)[1]	(AS-weak, ≥5000)[1]	(AS-weak, ≥5000)[1]
**MAFG**	NS	NS	AS-weak
**ATF2**	NS	NS	≥5000
**ATF3**	NS	NS	NS
**ATF4**	**86.6** (94.8, 78.4, 18.5)[1]	(AS-weak, AS-weak*, 8.5)[1]	NI (NI, NI, NI)[1]
**ATF5**	(NS, NS, AS-weak)[1]	**420** (404.4, 453.9, 402.8)[1]	(2.9, 6.9, AS-moderate)[1]
**ATF6**	AS-weak	AS-weak	AS-weak
**ATF6B**	ND[1]	≥5000	≥5000
**CREBZF**	NS	AS-weak	**375**
**XBP1**	AS-weak	AS-weak	≥5000
**NFE2**	NS	NS	AS-weak
**NFE2L1**	NS	NS	NS
**NFE2L2**	NS	NS	NS
**NFE2L3**	NS	NS	NI
**CREB1**	NS	≥5000	**506**
**CREB3**	NS	NS	NS
**CREB3L1**	NI	AS-weak	AS-weak
**CREB3L3**	NS	AS-weak	AS-weak
**BACH1**	NS	AS-weak	AS-weak
**BACH2**	AS-weak	AS-weak	NI
**BATF**	≥5000	**382**	**136**
**BATF2**	≥5000	**509**	**76**
**BATF3**	AS-weak	**315**	**52.8**
**HLF**	NS	NS	AS-weak
**DBP**	NS	NS	AS-weak
**NFIL3**	NS	NS	NS
